# Notes on the scorpions (Arachnida, Scorpiones) from Xizang with the redescription of *Scorpiops jendeki* Kovařík, 2000 (Scorpiones, Euscorpiidae) from Yunnan (China)

**DOI:** 10.3897/zookeys.301.4608

**Published:** 2013-05-17

**Authors:** Zhiyong Di, Xiaobo Xu, Zhijian Cao, Yingliang Wu, Wenxin Li

**Affiliations:** 1 College of Life Sciences, Wuhan University, Wuhan, Hubei, 430072, China

**Keywords:** Scorpions, *Scorpiops*, taxonomy, checklist, key, Tibet, Xizang

## Abstract

Until now, there are 26 scorpion species of 7 genera of 5 families recorded in Xizang (China). Xizang Autonomous Region (Tibet) is the scorpion biodiversity richest area in China (53 scorpion species of 12 genera of 5 families), also the highest altitude habitat of scorpions in the world. We present information of type specimens, an identification key of the scorpion species from Xizang, the distribution, updated feature pictures, and discussion on the disputed species. The redescriptions of *Scorpiops jendeki* Kovařík, 2000 (Yunnan) and *Scorpiops tibetanus* Hirst, 1911 (Xizang), comments and feature figures of species of genus *Scorpiops* are provided for identification.

## Introduction

Xizang (Tibet) Autonomous Region is located in southwest China (26°52'–36°32'N, 78°24'–99°06'E), about 1,228,400 km^2^ (≈12.5% of China), famous as the “Roof of the world”. Xizang facing Xinjiang and Qinghai to the north, Sichuan and Yunnan to the east, while India, Myanmar, Bhutan, Sikkim, Nepal and Kashmir to the south and west (Bai, 2004). It is the main part of the Qinghai-Tibet Plateau, with an average elevation of more than 4,000 m, its central part above 4,500 m.

*Scorpiops tibetanus* Hirst, 1911 (Euscorpiidae) was the first scorpion species established by Xizang (China) specimens. Hirst (1911) described this new species by comparing it with some relatives briefly. Almost ninety years later, Kovařík (2000a and b) reported 2 new species: *Chaerilus tryznai* Kovařík, 2000 (Chaerilidae); *Scorpiops margerisonae* Kovařík, 2000 and 1 new record: *Scorpiops hardwickii* (Gervais, 1843). Zhu, Qi and Song (2004) summarized the historical scorpion records in China: totally 19 species and subspecies of scorpion s belonging to 9 genera and 5 families according to the relevant literatures, and presented the distribution information on species from Xizang: *Heterometrus petersii* (Thorell, 1876) (Scorpionidae) and *Scorpiops petersii* Pocock, 1893. From 2005, much more work on Xizang was finished. Kovařík (2005) described *Euscorpiops novaki* Kovařík, 2005 (Euscorpiidae). After scientific expedition, Qi, Zhu and Lourenço (2005) published the first comprehensive report of scorpions from Xizang, discovered 6 new species belonging to Chaerilidae (*Chaerilus*) and Euscorpiidae (*Euscorpiops* and *Scorpiops*): *Scorpiops atomatus* Qi, Zhu & Lourenço, 2005; *Scorpiops langxian* Qi, Zhu & Lourenço, 2005; *Scorpiops luridus* Qi, Zhu & Lourenço, 2005; *Scorpiops pococki* Qi, Zhu & Lourenço, 2005; *Euscorpiops karschi* Qi, Zhu & Lourenço, 2005; and *Chaerilus tessellatus* Qi, Zhu & Lourenço, 2005. Lourenço, Qi and Zhu (2005) identified *Mesobuthus songi* Lourenço, Qi & Zhu, 2005 (Buthidae), and *Heterometrus tibetanus* Lourenço, Qi & Zhu, 2005. Lourenço and Qi (2006) established of a new genera by specimens from Xizang: *Tibetiomachus* Lourenço & Qi, 2006 (Hemiscorpiidae) and new species: *Tibetiomachus himalayensis* Lourenço & Qi, 2006. Bastawade (2006) reported 2 new species and 4 new records: *Chaerilus dibangvalleycus* Bastawade, 2006; *Chaerilus pictus* (Pocock, 1890); *Chaerilus tricostatus* Pocock, 1899; *Euscorpiops asthenurus* (Pocock, 1900); *Euscorpiops kamengensis* Bastawade, 2006; *Scorpiops leptochirus* Pocock, 1893 by the specimens from South Xizang (China). Lourenço and Zhu (2008) discovered a new species belonging to *Isometrus* (Buthidae): *Isometrus (Reddyanus) tibetanus* Lourenço & Zhu, 2008, at the same time, *Isometrus* was a new recorded genus to Xizang. Zhu, Han and Lourenço (2008) summarized the chaerilid scorpions of China, and provided the redescriptions for *Chaerilus tessellatus* Qi, Zhu & Lourenço, 2005 and *Chaerilus triznai* Kovařík, 2000; pointed out that *Chaerilus pictus* (Pocock, 1890) which was described by Qi et al. (2005) was misidentified, and described one new species: *Chaerilus conchiformus* Zhu, Han and Lourenço, 2008, all of them from Xizang. Di and Zhu (2009a and b) established 2 new species: *Scorpiops lhasa* Di & Zhu, 2009 and *Chaerilus mainlingensis* Di & Zhu, 2009 successively. Di and Zhu (2009c) described the male of *Euscorpiops karschi* firstly. Di et al. (2009) analysed the genus *Chaerilus* Simon, 1877 (Scorpiones: Chaerilidae) from China, described the female *Chaerilus tricostatus* Pocock, 1899 firstly (*Chaerilus assamensis* Kraepelin, 1913 was a wrong record in this paper). Di and Zhu (2010) provided the redescription of *Scorpiops margerisonae* Kovařík, 2000, and reported the female for the first time. Sun, Zhu and Lourenço (2010) accommodated *Mesobuthus songi* Lourenço, Qi & Zhu, 2005 in the genus *Hottentotta*, as a new combination *Hottentotta songi* (Lourenço, Qi & Zhu 2005). In the meantime, Teruel and Rein (2010) transferred *Mesobuthus songi* Lourenço, Qi & Zhu, 2005 to the genus *Hottentotta* (Buthidae) too. Recently, Kovařík (2012) reported five new species of genus *Chaerilus*, including a Xizang species *Chaerilus wrzecionkoi* Kovařík, 2012.

Until now, twenty-six scorpion species of seven genera and five families were recorded in Xizang, all of them distribute in south and the north shores of Yarlung Zangbo Jiang. All of the eight species of *Chaerilus* from China found in Xizang, 10 of 11 species of *Scorpiops* from China living in Xizang (others: *Scorpiops jendeki* Kovařík, 1994 found in Yunnan, one unnamed species of *Scorpiops* in Hubei see Di et al. 2011a), 4 of 12 species of *Euscorpiops* from China found in Xizang (other 8 species in Yunnan; see Di et al. 2011b). The scientific expedition investigation of some areas of China has been finished basically which reflected in the papers (Qi et al. 2005; Shi et al. 2007; Zhang and Zhu, 2009; Di et al. 2009, 2010, 2011a and b, 2012; Sun and Sun, 2011). Followed these reports, Xizang is the richest area in China in scorpion diversity.

## Material and methods

Illustrations and measurements were produced using a Motic K-700L stereomicroscope with an Abbe drawing device and an ocular micrometer. Measurements follow Sissom (1990), and are given in mm. Trichobothrial notations follow Vachon (1974) and morphological terminology mostly follows Hjelle (1990). Terminology of metasomal carination follows Vachon (1952), Prendini (2000) and Soleglad and pedipalp chela carinae follow Sissom (2001) for. FKCP: private collection of F. Kovařík, Prague, Czech Republic; MHBU: Museum of the College of Life Sciences, Hebei University, Baoding, China; MNHN: Muséum national d’Histoire naturelle, Paris, France; NCZS: National Collections, Zoological Survey of India, Kolkata, India. NMPC: National Museum (Natural History), Prague, Czech Republic.

## Taxonomy

### Family Buthidae C. L. Koch, 1837

Buthidae: Fet & Lowe, 2000: 54–57; Soleglad & Fet, 2003: 89–91.

### Genus *Hottentotta* Birula, 1908

*Hottentotta*: Fet and Lowe, 2000: 134–135; Kovařík, 2007: 2–3, 8–10. Sun et al., 2010: 40.

#### Hottentotta songi

(Lourenço, Qi & Zhu, 2005)

http://species-id.net/wiki/Hottentotta_songi

Mesobuthus songi Lourenço, Qi & Zhu, 2005: 3–8, figs 1–17, tab. 1.
Hottentotta songi : Teruel & Rein, 2010: 7; Sun et al., 2010: 40–12, figs 25–29.

##### Type specimens.

Holotype, male; Paratypes, 9 males and 9 females, China, Xizang, south region of Pulan, low valley of the river Kongque He, near to the border with Nepal, 7/1931. Male holotype, 7 male and 8 female paratypes deposited in MNHN. 2 male and 1 female paratypes deposited in MHBU.

##### Distribution.

Burang County (Pulan Xian) (China).

### Genus *Isometrus* Ehrenberg, 1828

*Isometrus*: Fet & Lowe, 2000: 146; Kovařík, 2003: 1–2, figs 1–8, tab. 1.

### Subgenus *Reddyanus* Vachon, 1972

*Isometrus (Reddyanus)*: Fet & Lowe, 2000: 151; Kovařík, 2003: 5.

#### Isometrus (Reddyanus) tibetanus

Lourenço & Zhu, 2008

http://species-id.net/wiki/Isometrus_tibetanus

Isometrus (Reddyanus) tibetanus Lourenço & Zhu, 2008: 268–270, figs 14–26, 32, tab. 1.

##### Type specimens.

Holotype, male, China, Xizang Region of Chesu (?), 10/1970, Lindberg leg., deposited in MHBU.

##### Distribution.

Chesu (?, China).

### Family Chaerilidae Pocock, 1893

Chaerilidae: Fet, 2000a: 323. Kovařík, 2000a: 40–41; Soleglad & Fet, 2003: 92.

### Genus *Chaerilus* Simon, 1877

*Chaerilus*: Fet, 2000a: 323; Kovařík, 2000a: 38; Kovařík, 2005: 1; Qi, Zhu & Lourenço, 2005: 29; Lourenço & Zhu, 2008: 462.

#### Chaerilus conchiformus

Zhu, Han & Lourenço, 2008

http://species-id.net/wiki/Chaerilus_conchiformus

Chaerilus pictus : Qi, Zhu & Lourenço, 2005:34–38, figs 126–144.
Chaerilus conchiformus Zhu, Han & Lourenço, 2008: 38–44, figs 1–21, tab. 1.

##### Type specimens.

Holotype, female, China, Xizang, Nyingchi County, Bayi Town, 29°41'N, 94°21'E, 17/8/2002, Ming-Sheng Zhu leg. (Ar.–MHU–XZ0201); Paratype, 1 female juvenile, China, Xizang, Nyingchi County, Bayi town, 6/8/2003, Feng Zhang leg. (Ar.–MHU–XZ0202); Paratypes, 6 females, China, Xizang, Nyingchi County, Baishuwang Town, 29°34'N, 94°30'E, 7/2006, Ming-Sheng Zhu, Xiao-Feng Yang and Long Liu leg. (Ar.–MHU–XZ0601-0606); Paratype,1 male, China, Xizang, Mainling County, Pai Town, 29°12'N, 94°06' E, 30/7/2006, Zhu Ming-Sheng, Yang Xiao-Feng and Liu Long leg. (Ar.–MHU–XZ0102) (deposited in MHBU).

##### Habitat.

Under the stones in the farmland (highland barley) and forest (cypress).

##### Distribution.

Mainling County and Nyingchi County (China).

#### Chaerilus dibangvalleycus

Bastawade, 2006

http://species-id.net/wiki/Chaerilus_dibangvalleycus

Chaerilus dibangvalleycus Bastawade, 2006: figs 1–16.

##### Type specimens.

Holotype, male; Paratypes, 5 females, 5 males and 2 young ones, China, Xizang, Dibangvalley District, Mayodia, 1800 Mts (deposited in NCZS).

##### Other materials reported.

3 males and 4 females, 15/9/1991, D. B. Bastawade leg.; 1 male, 16/9/1991, K.Alia leg.; 2 males and 1 female and 2 young ones, 17/9/1991, D. B. Bastawade leg.

##### Distribution.

Mêdog County (China).

#### Chaerilus mainlingensis

Di & Zhu, 2009

http://species-id.net/wiki/Chaerilus_mainlingensis

Chaerilus mainlingensis Di & Zhu, 2009a: 97–102, figs 1–16.

##### Type specimens.

Holotype, female, China, Xizang, Mainling County, the Estate of Gongbuwang, 12/7/2008, Zhi-Yong Di and Guo-Dong Ren leg. (Ar.-MHU-XZML0801); 1 female paratype, same data as holotype (Ar.-MHU-XZML0802) (deposited in MHBU).

##### Habitat.

Under the stones of mixed forest.

##### Distribution.

Mainling County (China).

#### Chaerilus pictus

(Pocock, 1890)

http://species-id.net/wiki/Chaerilus_pictus

Chaerilus pictus : Fet, 2000a: 327; Kovařík, 2000a: 53–54; figs 21–22, 39, 42–43, tabs 1–2; Lourenço & Bernard, 2010: figs 30–31.

##### Materials reported.

Specific locality see Bastawade, 2006.

##### Distribution.

South Xizang (China); (Assam) India; (Silhet) Bangladesh.

#### Chaerilus tessellatus

Qi,Zhu & Lourenço, 2005

http://species-id.net/wiki/Chaerilus_tessellatus

Chaerilus tessellatus Qi, Zhu & Lourenço, 2005: 30, 34, figs 109–125; Zhu, Han & Lourenço, 2008: 44–47, figs 30–44, tab. 1.

##### Type specimens.

Holotype, female, China, Xizang, Mêdog County, Beibeng Town, 29°02'N, 95°03'E, 22/8/2003, Feng Zhang leg. (MHBU, Ar.–MHU–XZ0301); 2 female paratypes, China, Xizang, Bomi County, 29°08'N, 95°07'E, 14/8/2002, Ming-Sheng Zhu leg. (MHBU, Ar.–MHU–XZ0203; another deposited in MNHN); 1 female paratype, China, Xizang, Mêdog County, 108K-8K, 17/8/2003, Feng Zhang leg. (MHBU, Ar.–MHU–XZ0302).

##### Other materials reported.

1 female, China, Xizang, Bomi County, Mt. Sela, 3/8/2002, Ming-Sheng Zhu leg. (MHBU, Ar.–MHU–XZ0204); 2 femalejuveniles. China, Xizang, Nyingchi County, Dongjiu villige, 21/9/2007, Fu-Ming Shi leg. (MHBU, Ar.–MHU–XZ0401-02).

##### Distribution.

Bomê County (Bomi), Mêdog County and Nyingchi County (China).

#### Chaerilus tricostatus

Pocock, 1899

http://species-id.net/wiki/Chaerilus_tricostatus

Chaerilus tricostatus : Fet, 2000a: 327, Kovařík, 2000a: 61–62, figs 27–28, tabs 1–2; Di et al., 2009: 131–138; figs 1–18; tab. 1.

##### Materials reported.

3 females, 1 female immature and 3 juveniles, China, Xizang, Mêdog County, elevation 1146m, 29°20'N, 95°20'E, 14/8/2009, Liqing Fan leg. (Ar.-MWHU-XAMT0901–07; deposited in MWHU).

##### Distribution.

Mêdog County, South Xizang (China); (Assam) India.

#### Chaerilus tryznai

Kovařík, 2000

http://species-id.net/wiki/Chaerilus_tryznai

Chaerilus tryznai Kovařík, 2000a: 65–66, figs 32–33, tabs 1–2.
Chaerilus tryznai : Zhu, Han & Lourenço, 2008: 47–51, figs 45–60, tab. 1.

##### Type specimens.

Holotype, male; Allotype and Paratype (No. 1), 2 females; Paratypes Nos. 2–12, 10 females and 1 immature, China, Xizang, Bomi County, 29°52'N, 95°45'E, 3000m, M. Tryzna & O. Safranek, FKCP.

##### Other materials reported.

China, Xizang, Mêdog County, 29°02’N, 95°03’E, Hanmi Village, 11/8/2006, 1 female, Zhi-Shun Song leg. (Ar.–MHU–XZ0607) (in MHBU); China, Xizang, Mêdog County, Hanmi Village, 10/8/2006, 1 female, Zhi-Shun Song leg. Zhi-Shun Song leg. (Ar.–MHU–XZ0608) (deposited in MHBU).

##### Habitat.

Under the stones in the mixed forest.

##### Distribution.

Bomê County, Mêdog County (China).

##### Comments.

Five related species with close geographical distribution, *Chaerilus assamensis*: Kraepelin, 1913, *Chaerilus conchiformus*, *Chaerilus dibangvalleycus*, *Chaerilus mainlingensis*, and *Chaerilus tryznai*, all with 7–8 granulated cutting edges on the movable fingers of pedipalp (Bastawade, 2006; Di & Zhu, 2009; Kovařík, 2000a; Zhu, Han & Lourenço, 2008). *Chaerilus assamensis* was described by type specimen from Assam (India), its original description is poor (see Kraepelin, 1913). Kovařík (2000a: 69), who analysed the old reference, recorded three characters of *Chaerilus assamensis*: middle and lateral eyes present; 7–8 granulated cutting edges on the movable fingers of pedipalp; the anterior margin of carapace arched in males. Lourenço and Duhem (2010) thought *Chaerilus tryznai*, with few differences from *Chaerilus assamensis*, may prove to be conspecific. Both sexes of *Chaerilus conchiformus*, *Chaerilus dibangvalleycus* and *Chaerilus tryznai* have anterior margin truncated,but only females of *Chaerilus mainlingensis* have same anterior margin of carapace as *Chaerilus dibangvalleycus*. Except *Chaerilus assamensis* with poor information, other 4 species can be identified by the key provided in this paper.

#### Chaerilus wrzecionkoi

Kovařík, 2012

http://species-id.net/wiki/Chaerilus_wrzecionkoi

Chaerilus wrzecionkoi Kovařík, 2012b: 11–13, figs 62–77.

##### Type specimens.

Holotype, allotype and paratypes, 2 males and 2 females, China, Xizang, Tomi (Tangmai), 30 km W of Donjung, 2075 m a.s.l., 23/6/2007, leg. A. Wrzecionko; FKCP.

##### Distribution.

Tangmai (Tongmai?), Tomi (Bomê County?) (China).

##### Comments.

*Chaerilus wrzecionkoi* are closest with *Chaerilus mainlingensis* Di & Zhu, 2009 and *Chaerilus tryznai* Kovařík, 2000. Both have manus and patella of pedipalp narrower and longer than other congeneric species. *Chaerilus mainlingensis* has four distinct carinae on the seventh sternite; *Chaerilus wrzecionkoi* has the seventh sternite granulated but without carinae; manus of pedipalp in male narrow and long, chela length/width ratio in male higher than 3 in *Chaerilus tryznai* Kovařík, 2000, while manus of pedipalp in male robust and chela length/width ratio in adults lower than 2.6 in *Chaerilus wrzecionkoi* (see Kovařík, 2012).

### Family Euscorpiidae Laurie, 1896

Euscorpiidae: Fet & Sissom, 2000: 355; Soleglad & Fet, 2003: 105.

Scorpiopidae: Fet, 2000d: 487; Kovařík, 2000b: 154.

### Genus *Euscorpiops* Vachon, 1980

*Euscorpiops*: Fet & Sissom, 2000: 488. Kovařík, 2000b: 154; Kovařík, 2005: 1, 4; Kovařík, 2012a: 1, 3.

#### Euscorpiops asthenurus

(Pocock, 1900)

http://species-id.net/wiki/Euscorpiops_asthenurus

Euscorpiops asthenurus : Fet, 2000d: 488.
Scorpiops asthenurus : Kovařík, 2000b: 167, figs 15, 28, 31, tabs 1–3.

##### Other materials reported.

Specific locality see Bastawade, 2006.

##### Distribution.

South Xizang (China); Bhutan; (Assam, West Bengal, Sikkim) India.

#### Euscorpiops kamengensis

Bastawade, 2006

http://species-id.net/wiki/Euscorpiops_kamengensis

Euscorpiopskamengensis Bastawade, 2006: 454, 456, 457, figs 17–26.

##### Type specimens.

Holotype, male; Paratype, 1 female immature, China, South Xizang, West Kameng, 7 Kms of Bomdilla, Sara village, 2500 mts. D. B. Bastawade leg. 18/9/1991 (deposited in NCZS).

##### Distribution.

South Xizang (China).

#### Euscorpiops karschi

Qi, Zhu & Lourenço, 2005

http://species-id.net/wiki/Euscorpiops_karschi

Euscorpiops karschi Lourenço, Zhu & Qi, 2005: 25, figs 94–108. Di & Zhu, 2009b: 11–15, figs 1–27, tab. 1.

##### Type specimens.

Holotype, female, China, Xizang, Zayü district, Xia Zayü town (28°30'N, 97°00'E), 8/8/2002, Ming-Sheng Zhu leg. (MHBU, Ar.-MHBU-XZZY0201). Paratypes: 2 females and 2 immature males, same data as holotype (one female in MHBU, Ar.-MHBU-XZZY0202); one female in MNHN, Ar.-MNBU-XZZY0203).

##### Other materials reported.

1 male, China, Xizang, Zayü district, Xia Zayü town (28°30’N, 97°00’E), 2/10/2007, Fu-Ming Shi leg. (MHBU, Ar.-MHBU-XZZY0701); 1female, China, Xizang, Zayü district, Shang Zayü town, 23/8/2005, Zhi-Shun Song leg. (MHBU, Ar.-MHBU-XZZY0501).

##### Distribution.

Zayü County (Zayü district, Chayu district) (China).

#### Euscorpiops novaki

Kovařík, 2005

http://species-id.net/wiki/Euscorpiops_novaki

Euscorpiops novaki Kovařík, 2005: 4, 6, figs 8, 11, 15–16, tab. 1.

##### Type specimens.

Holotype, male, China, Xizang, Bomi County (29°52' N, 95°45'E), ca 3000 m, 1988, P. Rojek leg., FKCP.

##### Distribution.

Bomê County (China).

### Genus *Scorpiops* Peters 1861

*Scorpiops*: Fet, 2000d: 491; Kovařík, 2000b: 162, 164, 166; Qi, Zhu & Lourenço, 2005: 2; Di & Zhu, 2009: 40; Di et al., 2011b, 1–2. Kovařík, 2009: 1.

#### Scorpiops atomatus

Qi, Zhu & Lourenço, 2005

http://species-id.net/wiki/Scorpiops_atomatus

Figures 1 –21 , Table 1

Scorpiops atomatus Qi, Zhu & Lourenço, 2005: 6–10, figs 16–31.

##### Type specimens.

Holotype, male, China, Xizang, Lang district (29.02°N, 93.08°E), 7-8/2004, Ai-Min Shi and Yi-Bin Ba leg. (MHBU). Paratypes: 3 females, 1 male, same data as holotype (2 females in MHBU, 1 female and 1 male in MNHN); 1male, China, Xizang, Chayu district, Xia Zayü town (28.4°N, 97.0°E), 7/8/2002, Ming-Sheng Zhu leg. (MHBU?); 2 females, China, Xizang, Lang district (29.02°N, 93.08°E), 20 August 2002, Ming-Sheng Zhu leg. (MHBU?); 1 male, China, Xizang, Gyaca district (29.1°N, 92.7°E), 21/8/2002, Ming-Sheng Zhu leg. (MHBU?); 1 male, 1female, 22 August 2002, other data same as above (MHBU?).

##### Distribution.

Gyaca County, Nang County (Lang district, Langxian district), Zayü County (China).

##### Comments.

Kovařík & Ahmed (2009: 10) provided a list of *Scorpiops hardwickii* (Gervais, 1843) “complex”, which included 12 species, containing *Scorpiops atomatus*. Di et al. (2011a) summarized the characters of *Scorpiops hardwickii* “complex” and excluded *Scorpiops atomatus* with the reasons as followed: (1) pectinal teeth count is 9–11 in *Scorpiops atomatus*, and 4–8 in *Scorpiops hardwickii* (Kovařík, 2000: 178); (2) ventral trichobothria on patella number is 9 in *Scorpiops atomatus*, and 6–8 in *Scorpiops hardwickii* (Kovařík, 2000: 176); (3) fulcra are present in *Scorpiops atomatus* but absent in *Scorpiops hardwickii*. In addition, *Scorpiops atomatus* has clearly thinner chela than *Scorpiops pococki* and *Scorpiops langxian*.

**Table 1.** Measurements (in mm) of *Scorpiops atomatus*, *Scorpiops langxian* and *Scorpiops luridus*.<br/>

**Species<br/>Contents**	***Scorpiops atomatus***		***Scorpiops langxian***		***Scorpiops luridus***	
**Sex**	**Male<br/> HT (MHBU)**	**Female<br/> PT (MHBU)**	**Male<br/> HT (MHBU)**	**Female<br/> PT (MHBU)**	**Male<br/> HT (MHBU)**	**Female<br/> PT (MHBU)**
Total length	42.1	40.4	63.0	58.5	86.7	75.1
Carapace:						
-Length	6.2	6.4	7.7	7.3	11.7	10.2
- Anterior width	3.7	4.0	4.3	4.0	5.0	4.1
- Posterior width	6.5	6.2	8.4	7.9	10.6	9.2
Metasomal segment I:						
- Length	2.4	2.3	4.1	3.9	4.3	4.1
- Width	2.4	2.4	3.8	3.5	4.5	4.1
- Depth	2.0	1.9	3.1	2.8	3.7	3.2
Metasomal segment II:						
- Length	2.9	2.6	4.7	4.3	4.9	4.2
- Width	2.1	2.1	3.6	3.2	4.0	3.7
- Depth	1.8	1.9	2.9	2.8	3.7	3.1
Metasomal segment III:						
- Length	3.1	2.9	5.2	4.9	5.7	5.1
- Width	2.0	2.0	3.3	3.1	3.8	3.2
- Depth	1.8	2.0	3.0	2.6	3.6	3.1
Metasomal segment IV						
- Length	3.6	3.1	5.7	5.5	7.8	6.4
- Width	1.8	1.9	3.2	2.9	3.6	3.1
- Depth	2.0	2.0	3.0	2.6	3.7	3.1
Metasomal segment V						
- Length	5.4	5.2	9.0	8.3	12.8	9.6
- Width	1.8	2.0	2.9	2.6	3.2	2.7
- Depth	1.8	1.9	2.9	2.4	3.3	2.8
Telson:						
- Length	5.9	5.5	8.8	7.4	12.8	10.2
- Width	2.0	1.9	3.7	3.0	4.6	4.0
- Depth	2.0	1.8	3.4	2.7	4.6	3.8
Pedipalp femur:						
- Length	5.6	5.6	6.1	6.1	10.2	8.9
- Width	2.6	2.4	2.6	2.7	3.8	3.7
- Depth	1.9	1.9	2.4	2.4	2.6	2.6
Pedipalp patella:						
- Length	5.1	5.3	6.0	5.3	9.6	8.3
- Width	2.8	2.7	3.1	3.1	4.5	4.1
- Depth	2.3	2.3	3.1	3.2	3.8	3.8
Chela						
- Length (chela)	10.0	9.9	11.2	11.5	23.5	20.0
- Length (manus)	6.1	6.2	6.6	6.7	12.0	10.2
- Width	4.4	4.2	6.2	5.8	7.3	6.4
- Depth	3.0	2.8	4.6	4.2	5.7	4.6
Movable finger-Length	5.9	5.9	7.4	7.3	11.5	9.8
Pectinal teeth (left/right)	10/11	9/9	8/8	6/6	10/10	8/8

**Figure 1.**
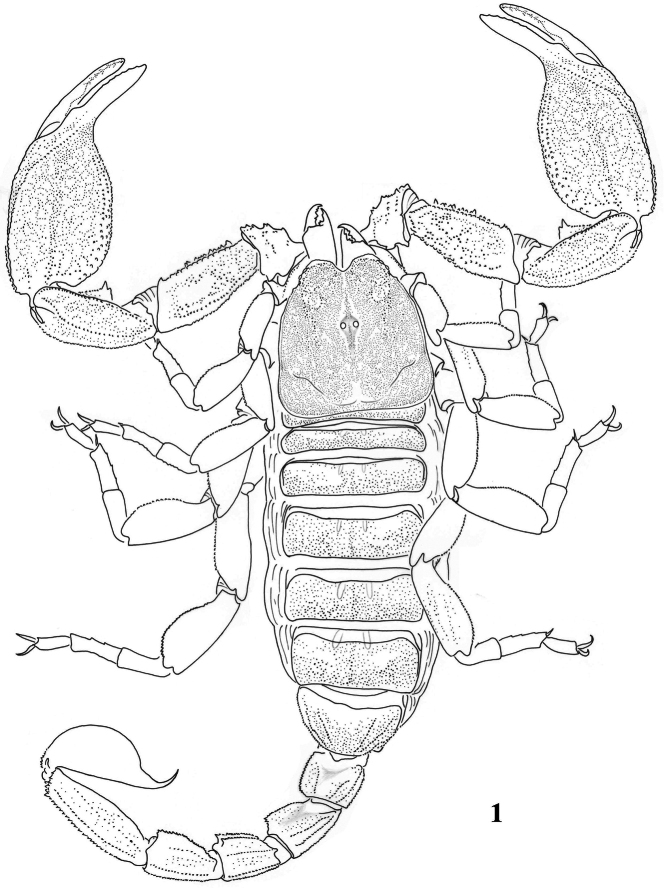
Habitus of *Scorpiops atomatus*, male, holotype, dorsal view.

**Figures 2–13.**
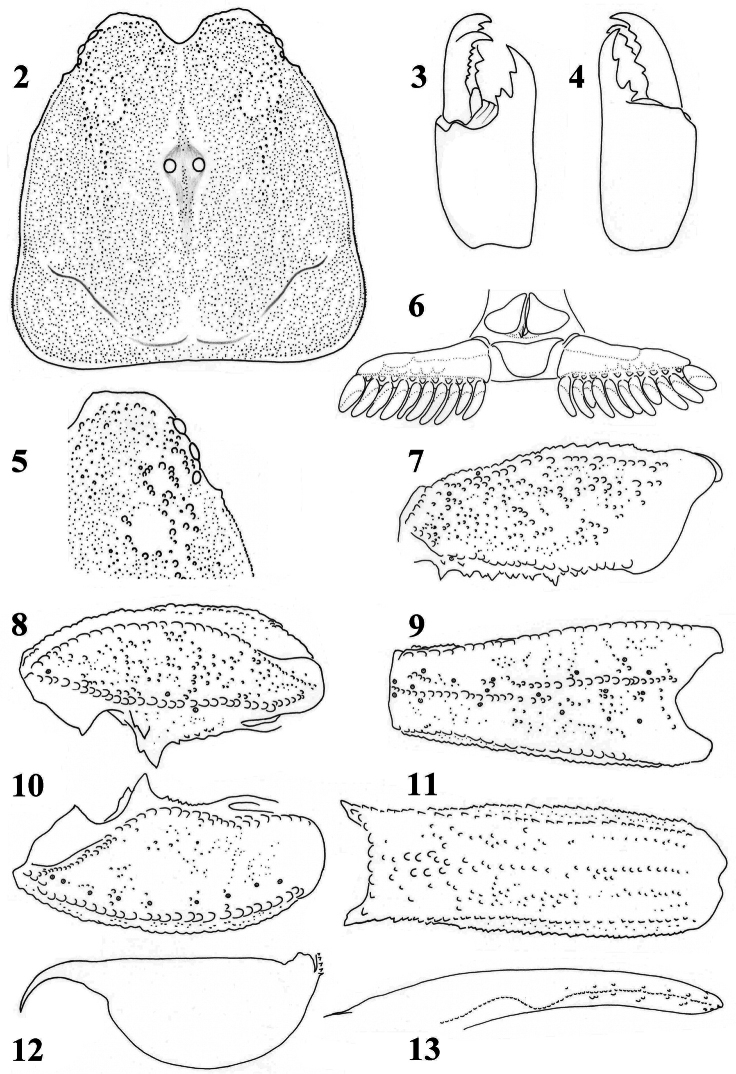
*Scorpiops atomatus*, male, holotype. **2** Carapace **3–4** Chelicera, dorsal and ventral aspects **5** Lateral eyes **6** Genital operculum and pectines **7** Femur dorsal aspect **8–10** Patella dorsal, external and ventral aspects **11** Metasomal segment V, ventral aspect **12** Telson, lateral aspect **13** Dentate margin of movable finger, showing rows of granules.

**Figures 14–17.**
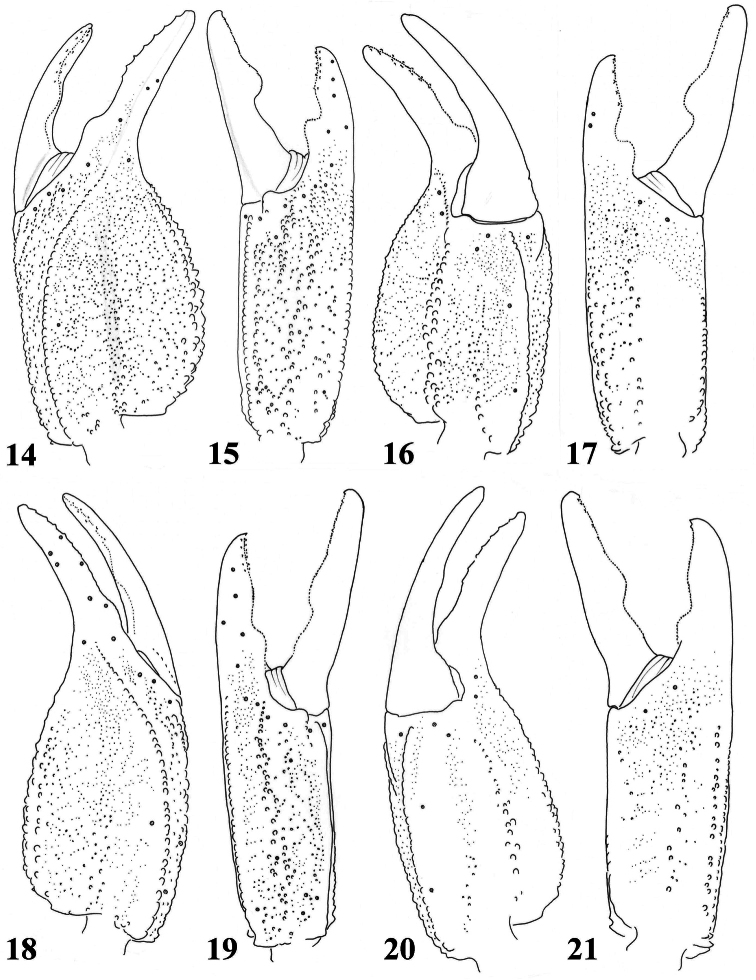
*Scorpiops atomatus*, male, holotype. Chela (left) dorsal, external, ventral and internal aspects **18–21** *Scorpiops atomatus*, female, paratype. Chela dorsal, external, ventral and internal aspects.

#### Scorpiops hardwickii

(Gervais, 1843)

http://species-id.net/wiki/Scorpiops_hardwickii

Scorpiops hardwickii : Kovařík, 2000b: 175–179, figs 14, 46, 56, 57.
Scorpiops hardwickii hardwickii :Fet, 2000d: 492.

##### Materials reported.

1 male, 3 females and 3 juveniles, China, Xizang, Nyainqentangha Mts, Lhasa, 3800m, V. Major leg. FKCP.

##### Distribution.

Lhasa, Xizang (China); (Himachal, Uttar, Jammu, Kashmir, Punjab) India; Nepal; Pakistan.

##### Comments.

The list of *Scorpiops hardwickii* (Gervais, 1843) “complex”, provided by Kovařík & Ahmed (2009: 10), containing 12 species widely distributed in Asia. Di et al. (2011a) summarized the characters of *Scorpiops hardwickii* “complex”: (1) color red brown to dark brown; (2) total length about 45–80 mm in adults; (3) fingers of pedipalps very strongly flexed (curved) in males, slightly flexed (undulated) in females; (4) ventral trichobothria on patella number 6–8; (5) pectinal teeth number 4–9; (6) length/width ratio of chela about 1.8–2.1; (7) fulcra absent; (8) patella with two small spinoid granules on the internal aspect.

#### Scorpiops langxian

Qi, Zhu &Lourenço, 2005

http://species-id.net/wiki/Scorpiops_langxian

Figures 22 –42 , Table 1

Scorpiops langxian Qi, Zhu & Lourenço, 2005: 10–18, figs 32–46.

##### Type specimens.

Holotype, male, China, Xizang, Lang district (29°02’N, 93°08’E), 7-8/2004, Ai-Min Shi and Yi- Bin Ba leg. (MHBU); Paratypes 1 female, 1 male same data as holotype (MHBU); 1 female, China, Xizang, Nyingchi district (29°34’N, 94.30°E), Baishuwang town, 21/8/ 2003, Feng Zhang leg. (MNHN).

##### Distribution.

Nang County, Nyingchi County (China).

##### Comments.

Kovařík & Ahmed (2009: 10) provided a list of *Scorpiops hardwickii* (Gervais, 1843) “complex”, which contained 12 species, including *Scorpiops langxian*, and its features accord with the summary of Di et al (2011a).

**Figure 22.**
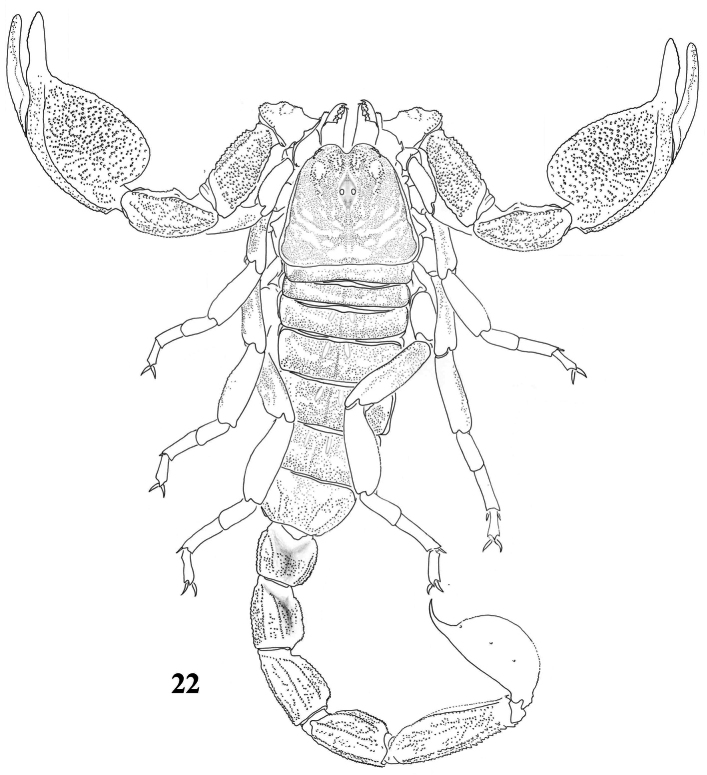
Habitus of *Scorpiops langxian*, male, holotype, dorsal view.

**Figures 23–34.**
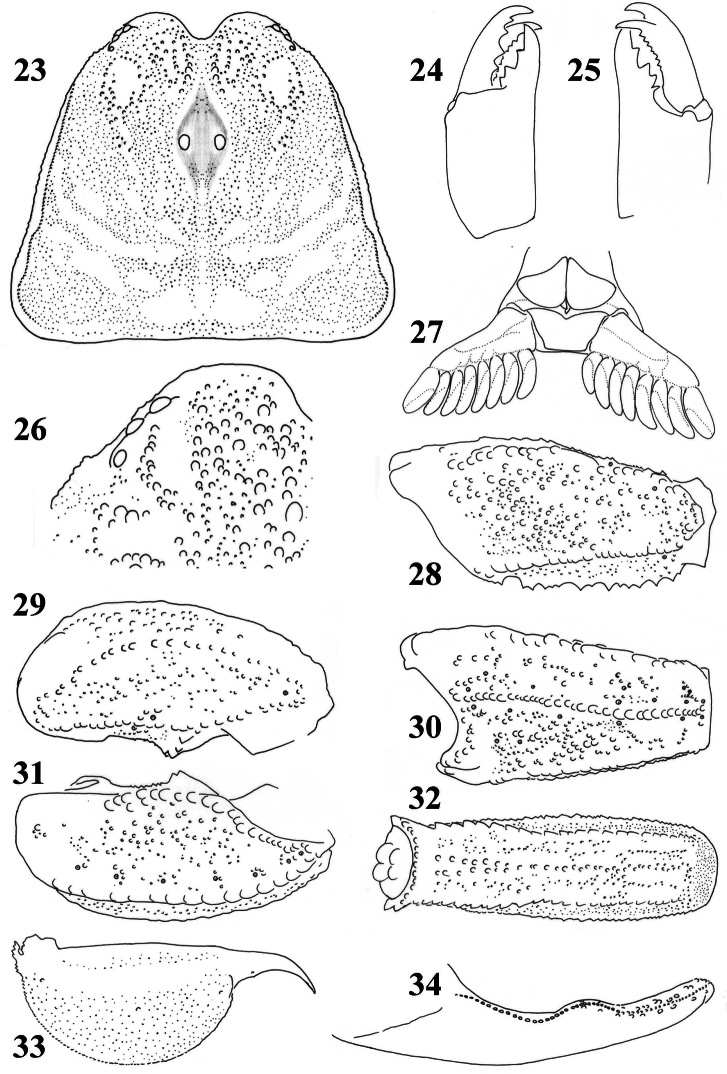
*Scorpiops langxian*, male, holotype. **23** Carapace **24–25** Chelicera, dorsal and ventral aspects **26** Lateral eyes **27** Genital operculum and pectines **28** Femur dorsal aspect **29–31** Patella dorsal, external and ventral aspects **32** Metasomal segment V, ventral aspect **33** Telson, lateral aspect **34** Dentate margin of movable finger, showing rows of granules.

**Figures 35–38.**
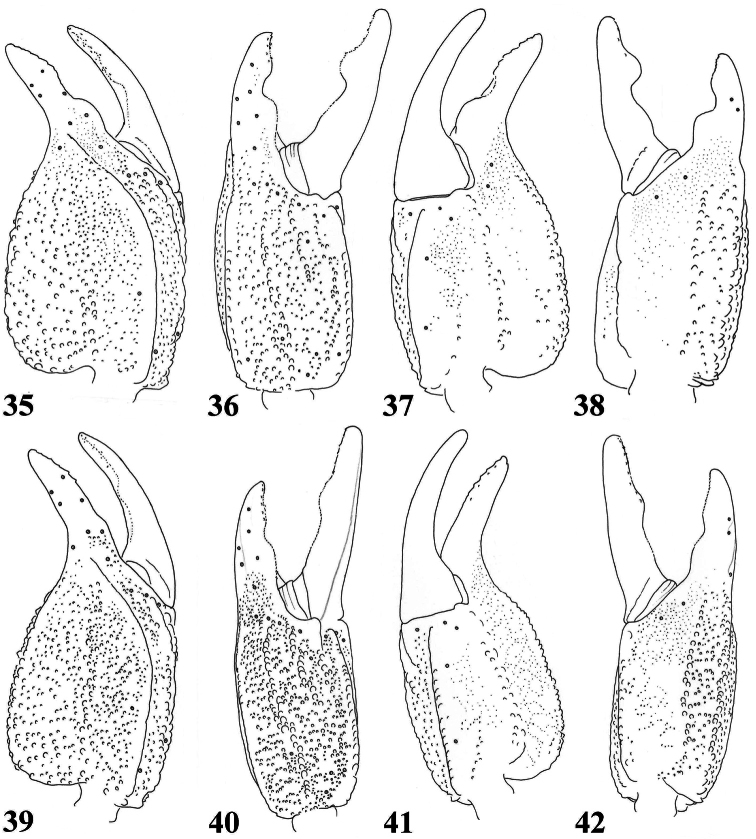
*Scorpiops langxian*, male, holotype. Chela dorsal, external, ventral and internal aspects **39–42** *Scorpiops langxian*, female, paratype. Chela dorsal, external, ventral and internal aspects.

#### Scorpiops leptochirus

Pocock, 1893

http://species-id.net/wiki/Scorpiops_leptochirus

Scorpiops leptochirus Pocock, 1893: Fet & Sissom, 2000b: 493.

##### Materials reported.

Specific locality see Bastawade, 2006.

##### Distribution.

South Xizang (China); Bangladesh; (Meghalaya, Assam) India.

#### Scorpiops lhasa

Di & Zhu, 2009

http://species-id.net/wiki/Scorpiops_lhasa

Scorpiops lhasa Di & Zhu, 2009c: 40–47, figs 1–33, tab. 1.

##### Type specimens.

Holotype, female, China, Xizang, Lhasa banlieue, elevation about 3700m, 10/7/2008, Zhi-Yong Di leg (Ar.-MHU-XZLS0801); paratypes: 1 female and 1 female juvenile, 2 males and 1 male juvenile, same data as holotype (Ar.-MHU-XZLS0802–0806) (deposited in MHBU).

##### Habitat.

Under the stones of barren mountain.

##### Distribution.

Lhasa (China).

#### Scorpiops luridus

Qi, Zhu & Lourenço, 2005

http://species-id.net/wiki/Scorpiops_luridus

Figures 43 –63 , Table 1

Scorpiops luridus Qi, Zhu & Lourenço, 2005: 2–6, figs 1–15.

##### Type specimens.

Holotype, male, China, Xizang, Lang district (29°02’N, 93°08’E), 2/8/2002, Ming-Sheng Zhu leg. (deposited in MHBU). Paratypes: 2 females, same data as holotype (One is deposited in MHBU, the other in MNHN).

##### Habitat.

Under the stones of barren mountain.

##### Distribution.

Nang County (China).

##### Comments.

*Scorpiops luridus* is the absolute offbeat member of *Scorpiops*: large body, pale yellow color, strong chelas and swollen telson. We checked other specimens (1 male and 1 female, from Shannan Prefecture, Xizang) and the type specimens, confirmed the distinctive color of this species not because of the immature age after molting.

**Figure 43.**
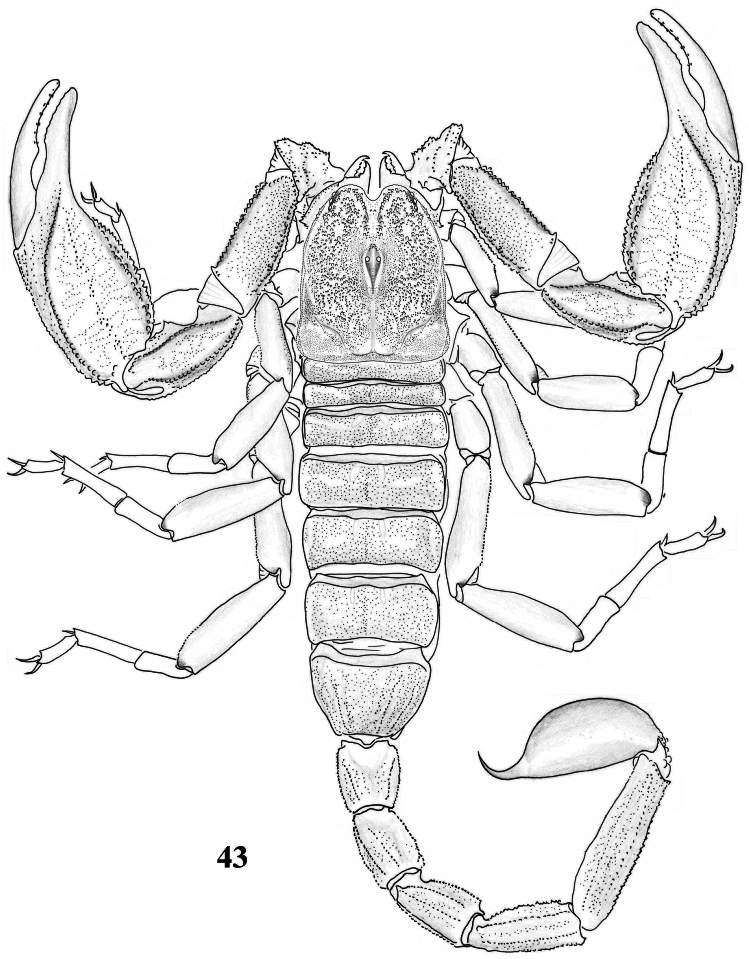
Habitus of *Scorpiops luridus*, male, holotype, dorsal view.

**Figures 44–54.**
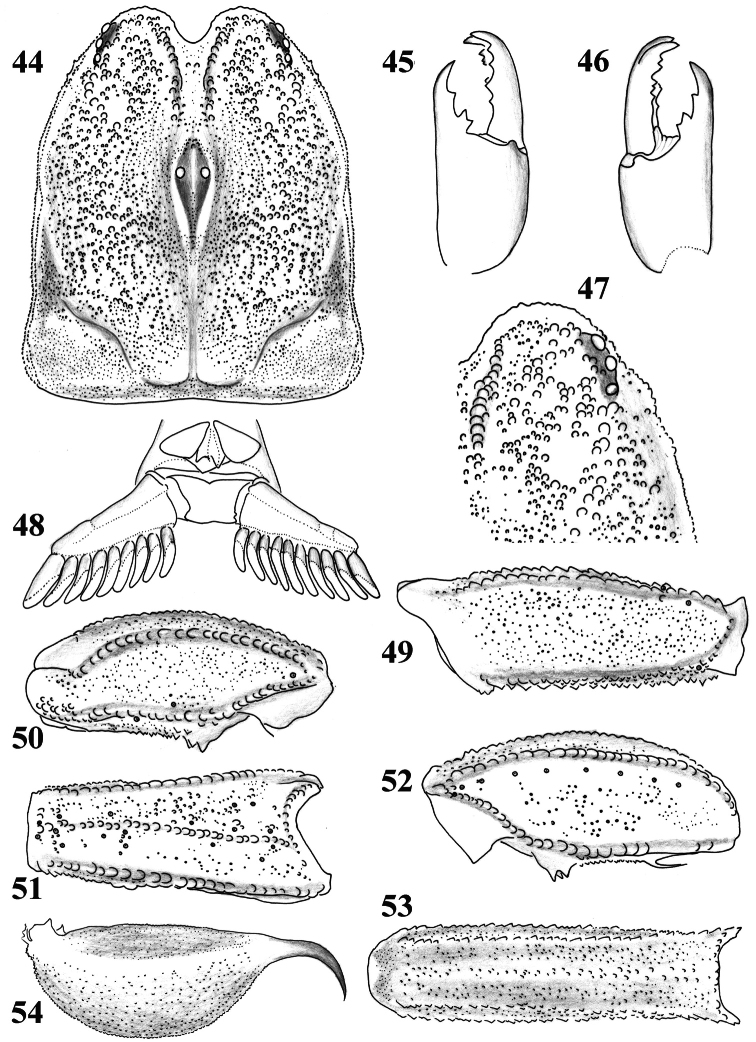
*Scorpiops luridus*, male, holotype. **44** Carapace **45–46** Chelicera, dorsal and ventral aspects **47** Lateral eyes **48** Genital operculum and pectines **49** Femur dorsal aspect **50–52** Patella dorsal, external and ventral aspects **53** Metasomal segment V, ventral aspect **54** Telson, lateral aspect.

**Figures 55–59.**
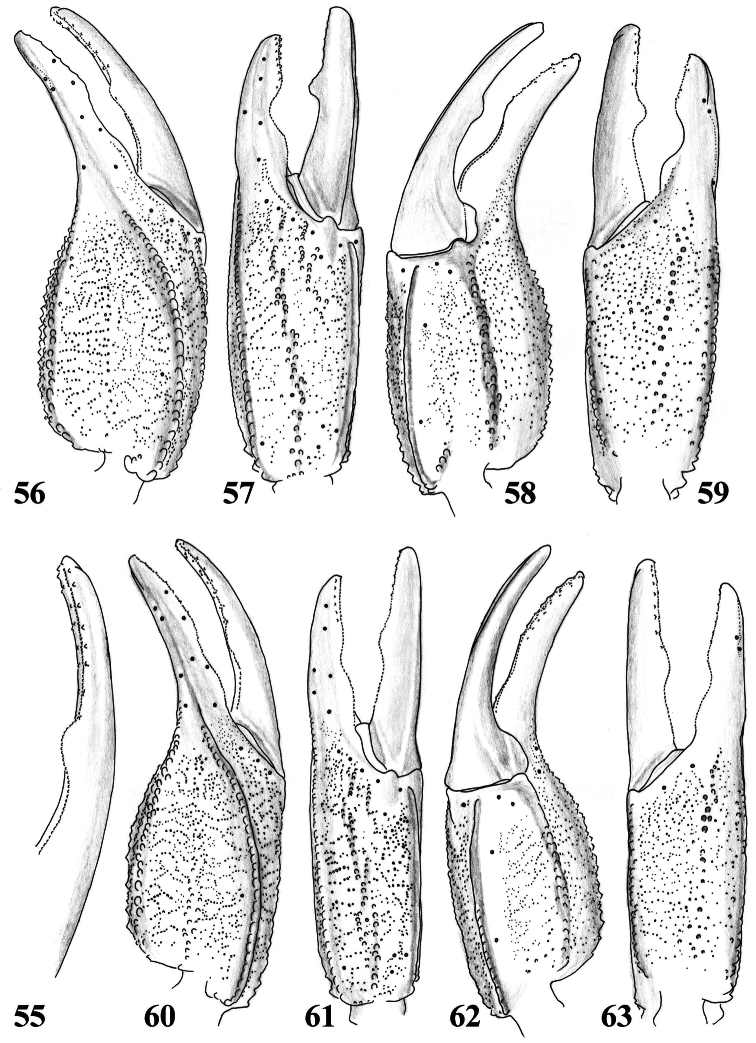
*Scorpiops luridus*, male, holotype. **55** Dentate margin of movable finger, showing rows of granules **56–59** Chela dorsal, external, ventral and internal aspects **60–63** *Scorpiops luridus*, female, paratype. Chela dorsal, external, ventral and internal aspects.

#### Scorpiops margerisonae

Kovařík, 2000

http://species-id.net/wiki/Scorpiops_margerisonae

Scorpiops margerisonae Kovařík, 2000b: 189, figs 66, 70, tabs 1–3; Di & Zhu, 2010: 1–8, figs 1–23, tabs 1–2.

##### Type specimens.

Holotype, male, China, Xizang, FKCP.

##### Other materials reported.

1 male, 1 female (Ar.-MHBU-XZLX060137, Ar.-MHBU-XZLX060138) and 7 juveniles., Langxian District, China, Xizang, 4/8/2006, leg. Ming-Sheng Zhu; 5 males (Ar.-MHBU-XZND060188, Ar.-MHBU-XZND060218, Ar.-MHBU-XZLX060238, Ar.-MHBU-XZLX060245, Ar.-MHBU-XZLX060246), 4 females (Ar.-MHBU-XZND060189, Ar.-MHBU-XZND0 60219, Ar.-MHBU-XZLX060220, Ar.-MHBU-XZLX06 0247), 1 female (imm.) (Ar.-MHBU-XZLX060248) and 5 juveniles, Naidong District, China, Xizang, 9/8/2006, leg. Ming-Sheng Zhu.

##### Habitat.

Found under stones.

##### Distribution.

Nang County, Nêdong County (Naidong district) (China).

##### Comments.

*Scorpiops margerisonae* was establishedby Kovařík (2000b) just using 1 male specimens. Its most important character provided by Kovařík (2000b) is the highest numer of pectinal teeth (12–13). Although the original description is poor, we can find another valuable information: *Scorpiops margerisonae* has a pair strong chelas with rectangular manus (with big granules in surface). Di & Zhu (2010) redescribed *Scorpiops margerisonae* and reported its female for the first time, and changed its pectinal teeth numer characters as a range 8–10 in females, 9–13 in males.

#### Scorpiops petersii

Pocock, 1893

http://species-id.net/wiki/Scorpiops_petersii

Table 2

Scorpiops petersii : Kovařík, 2000b: 192–194, figs 35, 42, tabs 1–3; Fet, 2000d: 494.

##### Distribution.

Xizang (China); Bhutan; (Assam, Himachal, Uttar, Kashmir, Meghalaya, Sikkim) India; Pakistan.

##### Comments.

*Scorpiops petersii* Pocock, 1893 has a simple original description. Kishida (1939: 45) recorded this species distributed in Xizang and Xikang (western Sichuan and eastern Tibet of China). Kovařík (2000b) examined the lectotype and many specimens but thought it is necessary to re-evaluate the characters used in distinguishing this species from others in the genus scorpiops (Kovařík, 2000b: 193). We cannot distinguish *Scorpiops petersii* with *Scorpiops hardwickii* (Gervais, 1843) “complex” by the diagnostic characters provided by Kovařík (2000b: 193): total length is up to 75mm; male has finger of pedipalps strongly flexed; 17 external (5 *eb*, 2 *esb*, 2 *em*, 4 *est*, 4 *et*) and 7, or rarely 6 or 8 ventral trichobothria on the patella; pectinal teeth number 4–9. We checked *Scorpiops* sp (1 adult and 1 immature females and 1 immature male and 1 juvenile, Lhasa, 4/7/2008, Zhiyong DI leg, kept in MHBU), its adult female: body length 80.1mm (Figs 85–101; Table 2), very strong; ventral trichobothria on patella number 7 (with other: rarely 6 or 8); pectinal teeth number 4-9; a swollen telson. Except the unusual body length can let us conjecture the specimens from Lhasa maybe *Scorpiops petersii*, all of other features shared by *Scorpiops hardwickii* and *Scorpiops petersii*. We noticed body length is an important character but it like the pectinal teeth number and patella ventral trichobothria number, all of them are some ranges and few exceptions are normal. We can’t confirm any of these characters in one species if checked just few specimens. Here, we add it to *Scorpiops hardwickii* (Gervais, 1843) “complex” group. We checked an immature female (locality is Uttaranchal, India; identified as *Scorpiops petersii* by Kovařík). And confirm the diagnosis of *Scorpiops petersii* as follows: (1) male chela length to width ratio about 2.6, and about 2.5 in female (see Kovařík, 2000b: tab. 1); (2) male has finger of pedipalps strongly flexed; (3) 17 external (5 *eb*, 2 *esb*, 2 *em*, 4 *est*, 4 *et*) and 7, or rarely 6 or 8 ventral trichobothria on the patella; (4) pectinal teeth number 4–9; (5) total length above 65mm. The first character is the key difference between *Scorpiops petersii* and *Scorpiops hardwickii* (Gervais, 1843) “complex” group.

**Table 2.** Measurements (in mm) of *Scorpiops petersii*, *Scorpiops* sp (Lhasa) and *Scorpiops tibetanus*.* Data from Kovařík, 2000b.<br/>

**Species<br/> Contents**	Scorpiops petersii*		***Scorpiops* sp<br/> (Lhasa)**	***Scorpiops tibetanus***	Scorpiops tibetanus*	
**Sex**	**Male<br/> LT (BMNH)**	**Female<br/> AT (NHMB)**	**Female<br/> (XZLS0801)**	**Female<br/> (XZSH0601)**	**Male HT<br/> (BMNH)**	**Female<br/> (FKCP)**
Total length	69.3	67..0	80.1	45.2	60.4	53.2
Carapace:						
-Length	8.8	8.3	10.2	5.7	7.5	7.5
- Anterior width			5.5	3.5		
- Posterior width	8.0	8.7	10.8	6.1	7.5	7.7
Metasomal segment I:						
- Length	3.5	3.2	4.3	2.7	3.6	2.7
- Width	3.5	3.6	4.4	2.7	3.9	3.1
- Depth			3.6	2.3		
Metasomal segment II:						
- Length	4.1	3.6	5.6	3.2	4.3	3.2
- Width	3.0	3.4	4.0	2.4	3.5	2.7
- Depth			3.6	2.1		
Metasomal segment III:						
- Length	4.4	4.1	6.1	3.4	4.7	3.6
- Width	2.8	3.3	3.7	2.3	3.4	2.7
- Depth			3.6	2.1		
Metasomal segment IV						
- Length	5.0	4.4	6.5	3.8	5.2	4.2
- Width	2.6	3.0	3.5	2.1	3.2	2.5
- Depth			3.5	2.0		
Metasomal segment V						
- Length	8.2	7.1	10.3	6.0	8.1	6.7
- Width	2.3	2.8	3.2	2.0	2.9	2.4
- Depth			3.4	1.8		
Telson:						
- Length	8.7	7.5	10.2	5.6	7.6	6.7
- Width			4.1	2.2		
- Depth			4.0	2.1		
Pedipalp femur:						
- Length	7.2	6.6	7.9	4.7	5.3	5.4
- Width	3.3	3.0	3.6	2.1	2.4	2.4
- Depth			3.3	1.9		
Pedipalp patella:						
- Length	7.2	7.0	8.2	4.5	5.8	5.8
- Width	3.4	3.1	3.5	2.7	2.5	2.5
- Depth			4.0	2.6		
Chela						
- Length (chela)	15.1	13.8	16.5	9.3	11.9	12.5
- Length (manus)			9.1	5.6		
- Width	5.8	5.5	7.5	4.3	5.9	5.1
- Depth			5.9	3.5		
Movable finger-Length	7.5	8.1	9.9	5.5	6.8	7.0
Pectinal teeth (left/right)	5/5	7/7	5/5	7/7	8/7	8/7

#### Scorpiops pococki

Qi, Zhu & Lourenço, 2005

http://species-id.net/wiki/Scorpiops_pococki

Figures 64 –84 , Table 3

Scorpiops pococki Qi, Zhu & Lourenço, 2005: 14–18, figs 47–61.

##### Type specimens.

Holotype, male, China, Xizang, Gyaca district (29°08'N, 92°43'E), 22/8/2002, Ming-Sheng Zhu leg. (MHBU); paratypes: 7 females and 4males, same data as holotype (1 female and 1 male in MNHN, the others in MHBU); 1 female, China, Xizang, Zayü district, Xia Zayü town (28°30'N, 97°00'E), 7/8/2002, Ming-Sheng Zhu leg.; 1male, China, Xizang, Ny-ingchi district (29°34'N, 94°30'E), 2/8/2002, Ming- Sheng Zhu leg., 2 females, 3 males, 17/8/2002, other data same as above; 3 females, China, Xizang, Nêdong district (29°11'N, 91°48'E), 15/8/2002, Ming-Sheng Zhu leg.; 1 male, China, Xizang, Xigazê (29°16'N, 88°51'E), 7/9/2002, Ming-Sheng Zhu leg.; 3 females, China, Xizang, Lhasa (29°39'N, 91°08'E), 23/8/2003, Feng Zhang leg. (deposited in MHBU).

##### Distribution.

Gyaca County, Nêdong County, Nyingchi County, Zayü County, Lhasa (China).

##### Comments.

Kovařík & Ahmed (2009: 10) provided a list of *Scorpiops hardwickii* (Gervais, 1843) “complex”, including *Scorpiops pococki*. *Scorpiops pococki*’s features accord with the summary of Di et al. (2011a). We provided the figures of the type specimen of *Scorpiops pococki* andother members from Xizang.

**Table 3.** Measurements (in mm) of *Scorpiops pococki*, and *Scorpiops jendeki*. * Data from Kovařík, 2000b.<br/>

**Species<br/> Contents**	***Scorpiops pococki***		*Scorpiops jendeki		***Scorpiops jendeki***	
**Sex**	**Male<br/> HT (MHBU)**	**Female<br/> PT (MHBU)**	**Male<br/> (FKCP)**	**Female<br/> HT (FKCP)**	**Female<br/> (YNLL0801)**	**Female<br/> (YNLL0802)**
Total length	40.0	45.7	32.2	42.1	22.8	20.8
Carapace:						
-Length	6.2	7.1	4.5	5.1	3.1	2.9
- Anterior width	3.6	4.1			1.6	16.
- Posterior width	6.3	7.2	4.8	5.9	3.3	3.2
Metasomal segment I:						
- Length	2.2	2.5	1.9	2.1	1.3	1.1
- Width	2.4	2.2			1.6	1.5
- Depth	2.0	2.2	2.4	2.6	1.3	1.3
Metasomal segment II:						
- Length	2.8	2.8	2.1	2.5	1.5	1.4
- Width	2.1	2.3	2.1	2.3	1.4	1.3
- Depth	1.9	2.0			1.2	1.1
Metasomal segment III:						
- Length	3.0	3.1	2.3	2.6	1.5	1.6
- Width	2.0	2.2	2.0	2.1	1.3	1.2
- Depth	2.0	2.1			1.1	1.1
Metasomal segment IV						
- Length	3.5	3.4	2.8	3.1	1.8	2.8
- Width	1.9	2.0	1.9	2.1	1.2	1.1
- Depth	2.0	2.2			1.1	1.0
Metasomal segment V						
- Length	5.4	5.8	4.5	5.4	2.8	2.7
- Width	1.9	2.0	1.9	2.0	1.2	1.1
- Depth	1.8	1.8			1.0	0.8
Telson:						
- Length	5.8	6.2	4.9	4.8	3.0	3.0
- Width	2.3	2.4			1.1	1.1
- Depth	2.1	2.2			1.0	0.9
Pedipalp femur:						
- Length	5.5	4.3	3.5	4.2	2.2	2.1
- Width	2.4	2.8	1.5	1.6	1.1	1.0
- Depth	1.9	2.0			1.0	0.9
Pedipalp patella:						
- Length	5.1	5.6	3.7	4.4	2.3	2.4
- Width	2.8	3.1	1.7	1.8	1.1	1.1
- Depth	2.6	2.8			1.1	1.0
Chela						
- Length (chela)	10.0	10.8	7.5	8.3	4.4	4.1
- Length (manus)	6.2	6.6			2.6	2.6
- Width	4.7	4.9	3.4	3..8	1.8	1.8
- Depth	3.3	3.5			1.4	1.2
Movable finger-Length	6.1	6.6	4.0	4.6	2.7	2.8
Pectinal teeth (left/right)	11/10	9/10	5/5	4/4	4/4	4/4

**Figure 64.**
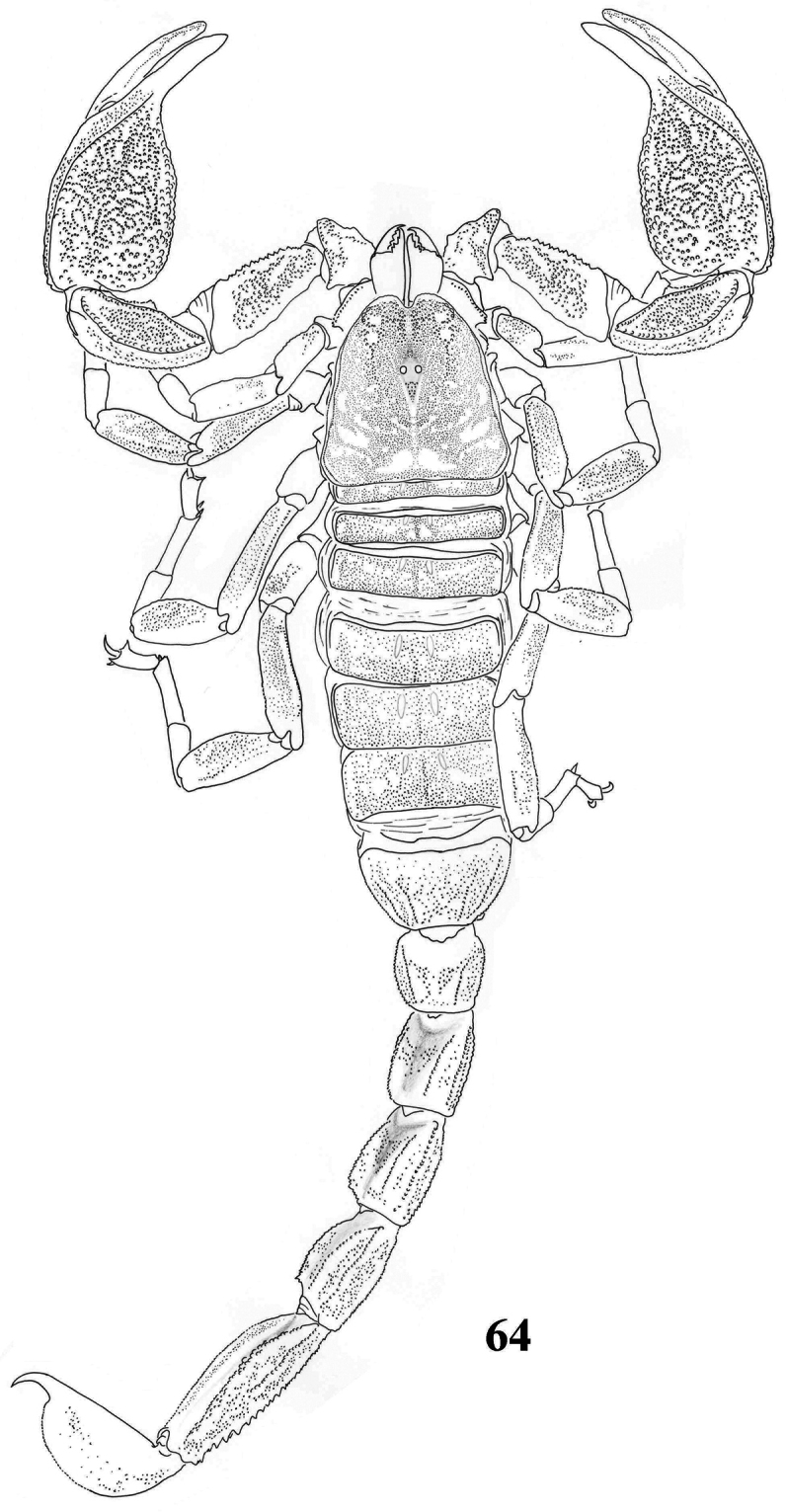
Habitus of *Scorpiops pococki*, male, holotype, dorsal view.

**Figures 65–76.**
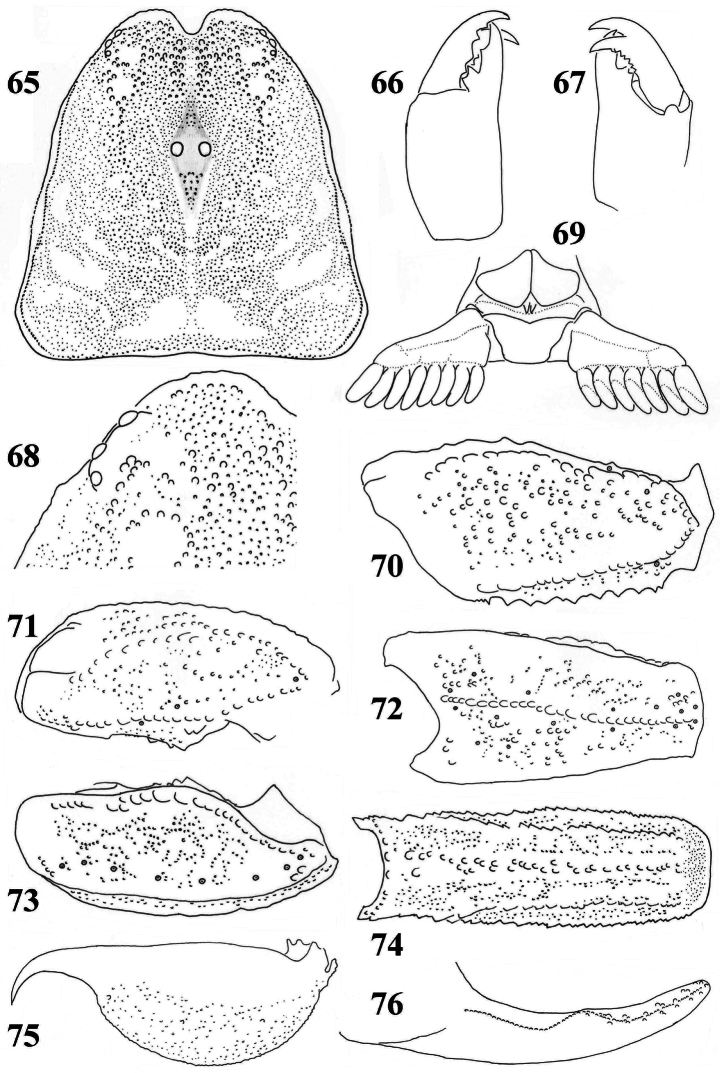
*Scorpiops pococki*, male, holotype. **65** Carapace **66–67** Chelicera, dorsal and ventral aspects **68** Lateral eyes **69** Genital operculum and pectines **70** Femur dorsal aspect **71–73** Patella dorsal, external and ventral aspects **74** Metasomal segment V, ventral aspect **75** Telson, lateral aspect **76** Dentate margin of movable finger, showing rows of granules.

**Figures 77–84.**
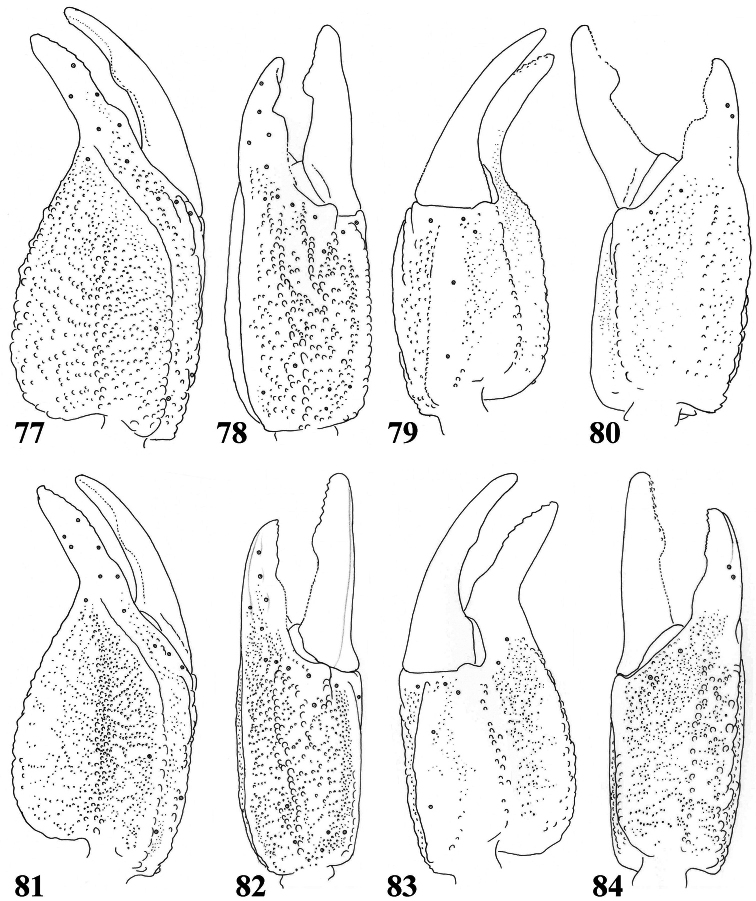
*Scorpiops pococki*. **77–80** male, holotype. Chela dorsal, external, ventral and internal aspects **81–84** female, paratype. Chela dorsal, external, ventral and internal aspects.

**Figure 85.**
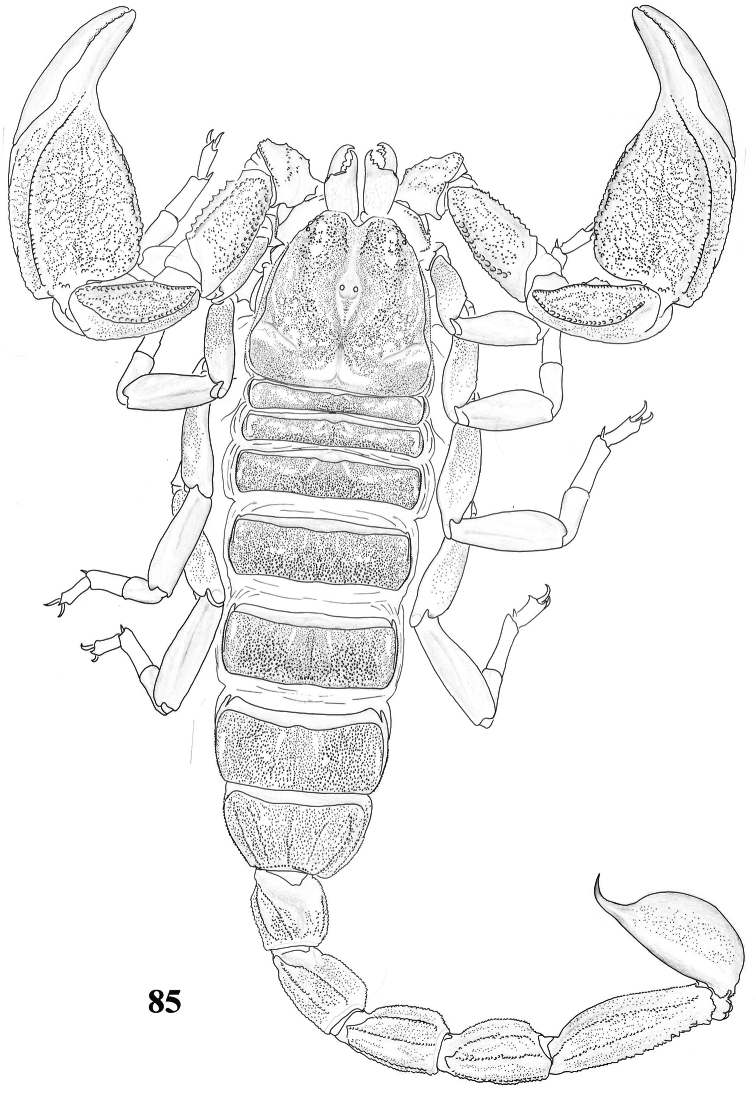
Habitus of *Scorpiops* sp. (*hardwickii* “complex”) from Lhasa, female, dorsal view.

**Figures 86–97.**
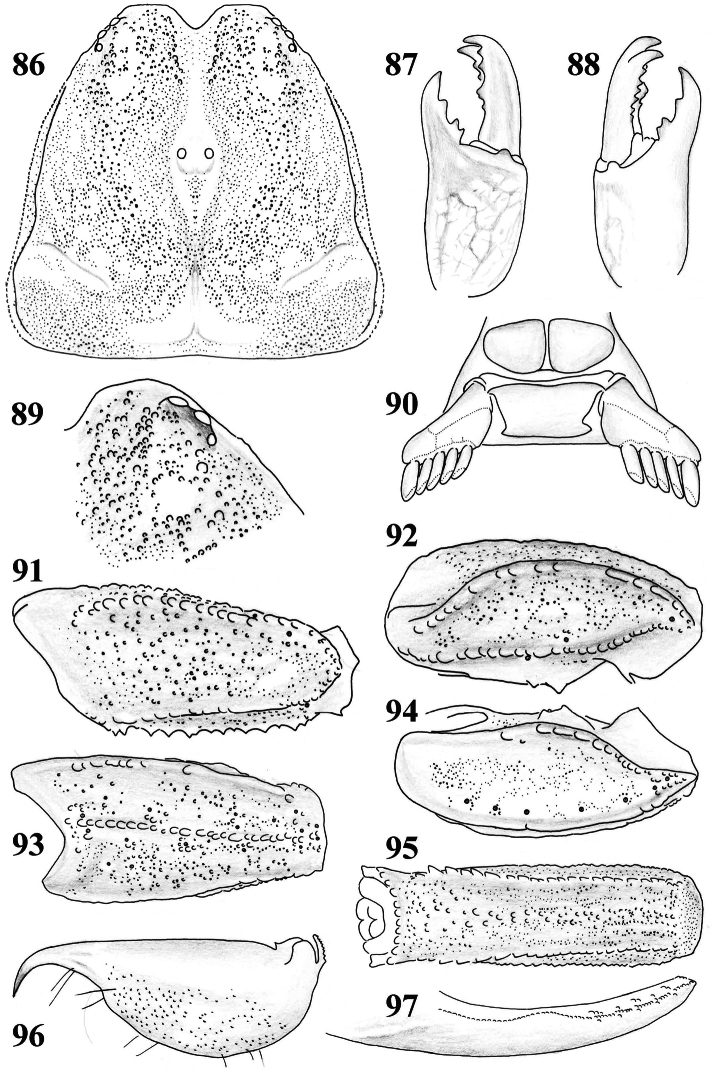
*Scorpiops* sp. (*hardwickii* “complex”) from Lhasa, female. **86** Carapace **87–88** Chelicera, dorsal and ventral aspects **89** Lateral eyes **90** Genital operculum and pectines **91** Femur dorsal aspect **92–94** Patella dorsal, external and ventral aspects **95** Metasomal segment V, ventral aspect **96** Telson, lateral aspect **97** Dentate margin of movable finger, showing rows of granules.

**Figures 98–101.**
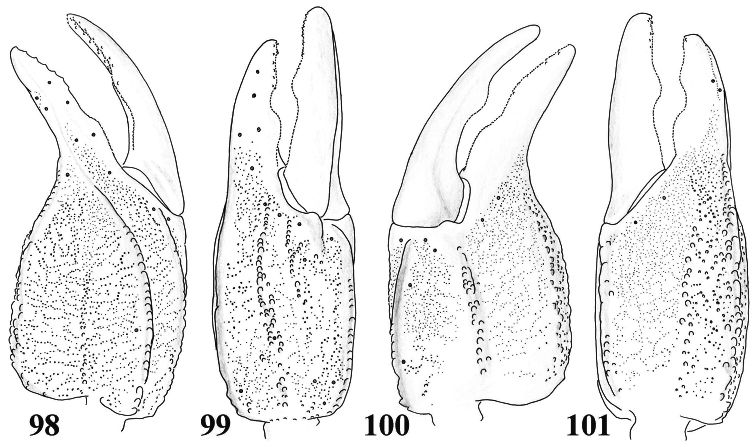
*Scorpiops* sp. (*hardwickii* “complex”) from Lhasa, female. Chela dorsal, external, ventral and internal aspects.

#### Scorpiops tibetanus

Hirst, 1911

http://species-id.net/wiki/Scorpiops_tibetanus

Figures 102 –118 , Table 2

Scorpiopstibetanus Hirst, 1911:472–473; Kovařík, 2000b: 197, figs 47, 68, 69, tab. 1–3; Fet, 2000d: 495.

##### Type locality.

China, Xizang, Tsangpo Valley, Chaksam Ferry..

##### Type material.

Holotype, male. L. A. Wadell leg. BMNH, No. 1911. 8. 10. 1.

##### Material examined.

1 female and 5 juveniles, China, Xizang, Shigatse City, around the Zhabulun Temple, 13/8/2006, Xiao-Feng Yang leg, (MHBU, Ar.- MHBU - XZSH0601–6).

##### Diagnosis.

Adult body length about 45–65 mm. Mainly color uniformly reddishblack. Male has finger of pedipalps more flexed and manus shorter and broader than the female. 17 external trichobothria (5 *eb*, 2 *esb*, 2 *em*, 4 *est*, 4 *et*) and 7–10 ventral trichobothria (usually 9) on the patella. Pectinal teeth number 5–11.

Comments. In Kovařík & Ahmed’s list of *Scorpiops hardwickii* (Gervais, 1843) “complex” (2009: 10): containing *Scorpiops tibetanus* Hirst, 1911. Hirst (1911) did not provide a detailed description except the brief comparison with *Scorpiops austerus* Hirst, 1911 (synonymized with *Scorpiops hardwickii* by Tikader & Bastawade, 1983: 418) and *Scorpiops crassimanus* Pocock, 1899 (synonymized with *Scorpiops hardwickii* by Kovařík, 2000b: 175). Kovařík (2000b) examined the holotype (male) of *Scorpiops tibetanus* and recorded some important information: (1) total length is 50–65mm; (2) male has finger of pedipalps more flexed and manus shorter and broader than the female; (3) 17 external trichobothria (5 *eb*, 2 *esb*, 2 *em*, 4 *est*, 4 *et*) and 7–10 ventral trichobothria (usually 9) on the patella; (4) pectinal teeth number 5–11. Di et al. (2011a) excluded *Scorpiops tibetanus* from Kovařík’s *Scorpiops hardwickii* “complex” as followed reasons: (1) ventral trichobothria on patella in S. *tibetanus* number 7–10 (usually 9, in one young out of 37 specimens, 7 on one side; Kovařík, 2000b: 196), 6–8 in *Scorpiops hardwickii* “complex”; (2) pectinal teeth number is 5–11 (usually 7–11) in *Scorpiops tibetanus*, 4–9 in *Scorpiops hardwickii* (usually 5–7).

##### Description.

(based on female specimens: Ar.- MHBU - XZSH0601).

*Coloration*: red brown mainly.

Carapace dark red brown. Median and lateral ocular tubercles black. Tergites mostly red brown to dark brown. Metasoma segments dark red brown to dark brown. Vesicle red brown with a reddish aculeus. Chelicerae yellow brown with fingers dark red brown gradually lighter toward the tip. Pedipalp femur and patella dark red brown, chela manus and fingers red brown. Legs red brown with yellow brown tarsi. Tarsal ungues yellowish brown. Sternum, genital operculum and sternites pale brown. Pectines yellowish.

*Morphology*. *Prosoma*: Carapace with sparse, coarse granules (Fig. 103); lateral furrow broad; anterior median furrow broad and moderately deep; posterior median furrow deep; margin behind lateral eyes with granules, other margins smooth. Median eyes situated anteriorly compared to center of carapace; three pairs of lateral ocelli, posterior smallest (Figs 103, 106). Median ocular tubercle with granules and a pair of big median eyes and a median furrow. Lateral ocular tubercle with some granules.

*Mesosoma*: Tergites sparsely covered with coarse and big granules, posterior part of tergites with bigger granules; tergites III–VI with a median carina; tergite VII with two pairs of lateral carinae (shaped by bigger granules); tergites margin smooth. Pectinal teeth count 7/7, fulcra present (Fig. 107). Sternum quinquangular. Genital operculum subtriangular. Sternites smooth and shiny; segment VII with 4 smooth ventral carinae and few granules.

*Metasoma*: Tegument coarse. Segments II to V longer than wide; segments I to V with respectively 10-8-8-8-7 carinae, segments II–IV with a pair of vestigial lateral carinae; all dorsal carinae crenulate, slightly stronger distally; segment V carinae with smaller granules dorsally and larger serration ventrally. Vesicle with few setae and granules. *Pedipalps*: Tegument coarse. Femur with external, dorsointernal, dorsoexternal, ventrointernal, ventroexternal and internal carinae granulated; tegument with scattered granules dorsally (Fig. 108) and smooth ventrally. Patella with dorsointernal, dorsoexternal, ventrointernal, ventroexternal and external carinae with big granules; two large spinoid granules present on the internal aspect; tegument with some granules. Trichobothrial pattern C, neobothriotaxic (Vachon 1974); patella with 17 external trichobothria (5 *eb*, 2 *esb*, 2 *em*, 4 *est*, 4 *et*), 9 ventral trichobothria (Figs 109–111). Chela with length/width ratio: 2.2–2.5 in adult females and 2.0 in male (holotype, Kovařík, 2000b: 161. tab. 1). Chela with dorsal marginal, external secondary, and ventrointernal carinae granulated (Figs 115–118); ventrointernal carina with some big granules; tegument with granules; female fingers scalloped with a pronounced lobe in the movable finger and a corresponding notch in fixed finger, lobe and corresponding notch reduced to absent in females. The male has fingers of pedipalps more flexed and manus shorter and broader than the female (Kovařík, 2000b: 196).

*Chelicerae*: Tegument smooth. Tibia smooth. Movable finger with 4 teeth on dorsal edge, 5teeth on ventral edge. Fixed finger with 3 teeth on dorsal edge (Figs 104, 105).

*Legs*: Tegument coarsely granular dorsally, except basitarsi and telotarsi, smooth ventrally. Trochanters with few setae. Femur dorsal surface with few small granules, external surface with a granular carina, internal surface with two granular carinae. Patella internally with a dentate carina. Tibia with few setae and small granules, without spurs. Basitarsi with some spinules, few setae and 2 lateral pedal spurs. Tarsi ventrally with one row of short spinules and few setae. Tarsal ungues curved and hook-like.

**Figure 102.**
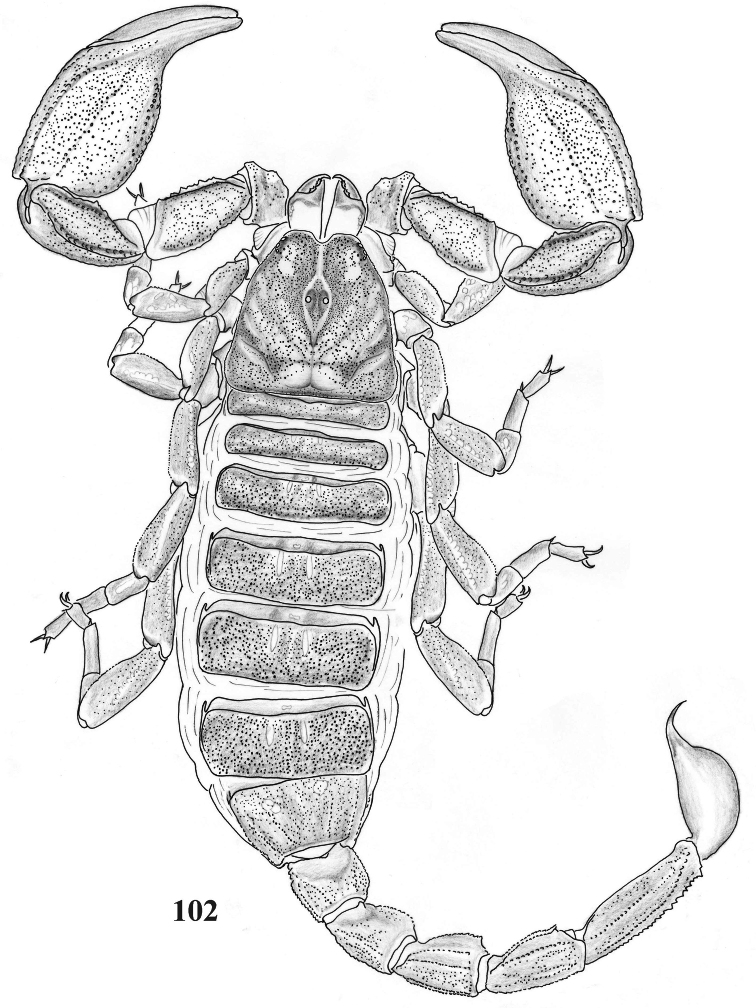
Habitus of *Scorpiops tibetanus* from Shigatse, female, dorsal view.

**Figures 103–113.**
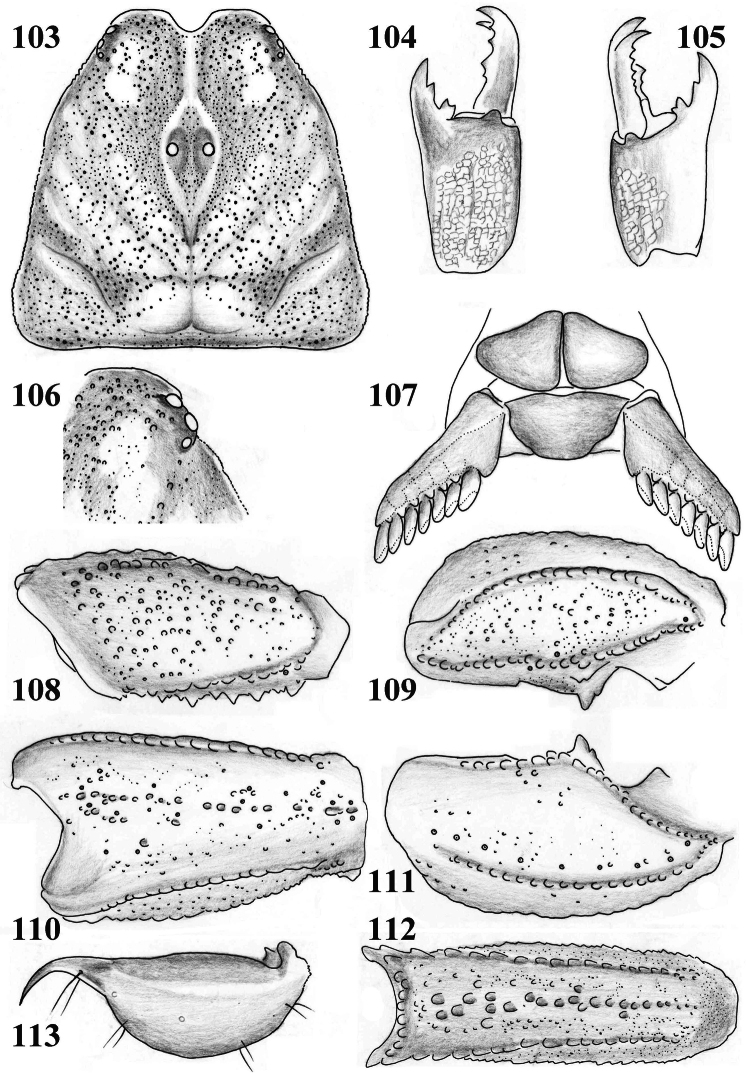
*Scorpiops tibetanus* from Shigatse, female. **103** Carapace **104–105** Chelicera, dorsal and ventral aspects **106** Lateral eyes **107** Genital operculum and pectines **108** Femur dorsal aspect **109–111** Patella dorsal, external and ventral aspects **112** Metasomal segment V, ventral aspect **113** Telson, lateral aspect.

**Figures 114–118.**
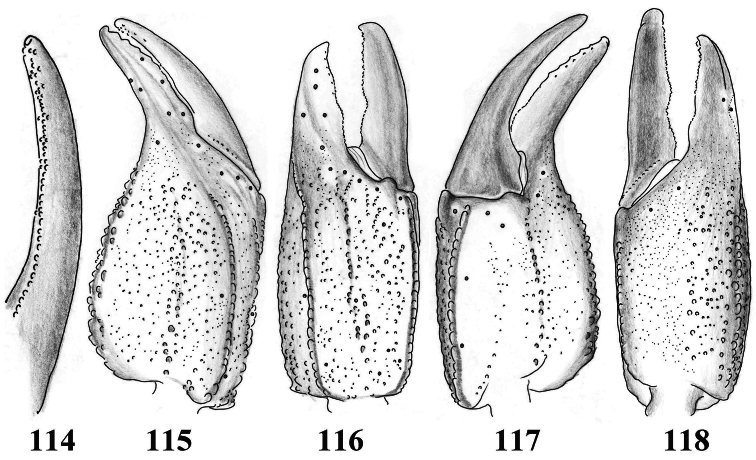
*Scorpiops tibetanus* from Shigatse, female. **114** Dentate margin of movable finger, showing rows of granules **115–118** Chela dorsal, external, ventral and internal aspects.

##### Variation.

Both sexes with coloration and morphology very similar to holotype. Sexual dimorphism: adult males, with more pronounced lobes on the movable fingers of the chela, and a more pronounced notch in the fixed finger and bigger pectinal teeth than females. Measurements in Table 2.

##### Ecology.

This species was collected from barren mountain. They were found under stones.

##### Distribution.

Tsangpo Valley and Xigazê (standard notation of Shigatse) (China).

### Family Hemiscorpiidae Pocock, 1893

Ischnuridae: Fet, 2000b: 383.

Liochelidae: Fet & Bechly, 2001: 1–2.

Liochelidae: Soleglad & Fet, 2003: 112–113.

Hemiscorpiidae: Soleglad, Fet & Kovařík, 2005: 1.

### Genus *Tibetiomachus* Lourenço & Qi, 2006

*Tibetiomachus* Lourenço & Qi, 2006: 291.

#### Tibetiomachus himalayensis

Lourenço & Qi, 2006

http://species-id.net/wiki/Tibetiomachus_himalayensis

Tibetiomachus himalayensis Lourenço & Qi, 2006: 291–294, figs 1, 2, 5–26.

##### Type specimens.

Holotype, female, China, Xizang, Guerla Mandhata, ≈4600 m, 7/1939 (Italian expedition leg) (deposited in MNHN).

##### Habitat.

In soil under rocks.

##### Distribution.

Guerla Mandhata (China).

### Family Scorpionidae Latreille, 1802

Scorpionidae: Fet, 2000c: 427–428. Soleglad & Fet, 2003: 113–114.

### Genus *Heterometrus* Hemprich & Ehrenberg, 1828

*Heterometrus*: Fet, 2000c: 431; Lourenço, Qi & Zhu, 2005: 9.

#### Heterometrus tibetanus

Lourenço, Qi & Zhu, 2005

http://species-id.net/wiki/Heterometrus_tibetanus

Heterometrus tibetanus Lourenço, Qi & Zhu, 2005: 10–14, figs 18–34,tab. 1.

##### Type specimens.

Holotype, male; Paratypes, 2 males, China, Xizang, south region of Pulan, low valley of the river Kongque He, near to the border with Nepal, 7/1931. Holotype and 1 paratype deposited in the MNHN. One paratype deposited in MHBU.

##### Distribution.

Burang County (China).

### Key to genera of Scorpiones from Xizang

**Table:** 

1	Orthobothriotaxic pattern type A; ventral aspect of leg tarsus with multiple irregular rows of setae, no trace of spinules; dorsal edge of cheliceral movable finger with *two* basal denticles; hemispermatophore is *flagelliform* (Buthidae)	2
–	Orthobothriotaxic pattern type B or C; ventral aspect of leg tarsus with or without irregular setal rows, spinules present medially; dorsal edge of cheliceral movable finger with a *single* basal denticle; hemispermatophore is either *fusiform* or *lamelliform*	3
2	Telson without subaculear tooth	*Hottentotta* Birula, 1908
–	Telson with subaculear tooth pointed or rounded (*Isometrus* Ehrenberg, 1828), Trichobothrium *db* on chela of pedipalp situated between trichobothria *et* and *est*. Males of most species have longer segments of metasoma and often also wider manus than females; segments of pedipals are of equal length in both sexes	Subgenus *Reddyanus* Vachon, 1972
3	Orthobothriotaxic pattern type B; sternum is *type 1*; hemispermatophore is *fusiform Chaerilus* Simon, 1877
–	Orthobothriotaxic pattern type C; sternum is *type 2*; hemispermatophore is *lamelliform* 4
4	Legs with two pedal spurs (though one or more pedal spurs are lost in many troglobitic species); ventral aspect of leg tarsus equipped with moderately developed setal pairs and/or median row of spinules (configuration 5, see Soleglad & Fet, 2003); paraxial organ without reflection of internobasal sperm duct (Chactoidea, see Soleglad & Fet, 2003, p. 92–93: Key to the superfamilies of parvorder Iurida); chelal fingers equipped with inner accessory denticles (IAD), outer denticles (OD) situated outside of median denticle (MD) row; major variable neobothriotaxy present, types Eu1 and Eu2; chelal palm is flat in appearance, carinae D3 and V2 essentially obsolete, angle formed by carinae D3: D4: D5 greater than 90° (Euscorpiidae, see Soleglad & Fet, 2003, p. 94: Key to the families of superfamily Chactoidea)	5
–	Legs with one pedal spur (retrolateral spur absent, though this character is reversed in some bothriurid genera); ventral aspect of leg tarsus equipped with pairs of large limbated socketed setae, median spinule row optional (configuration 4, see Soleglad & Fet, 2003); paraxial organ with reflection of internobasal sperm duct (Scorpionoidea, see Soleglad & Fet, 2003, p. 92–93: Key to the superfamilies of parvorder Iurida)	6
5	Tricho-bothrium *Eb_3_* on external surface of chela is located between trichobothria *Dt* and *Est*. Telson vesicle/aculeus juncture with annular ring	*Euscorpiops* Vachon, 1980
–	Trichobothrium *Eb_3_* on the external aspect of pedipalp chela located basally from trichobothrium Dt. Annular ring at vesicle/aculeus juncture absent	*Scorpiops* Peters, 1861
6	Median ocular tubercle of carapace shallow, not raised above carapace surface; 2 pairs of lateral eyes; telotarsus with lateral lobes truncated; *Est* located in middle of hand (Hemiscorpiidae, see Stockmann & Ythier, 2010: 201)	7
–	Median ocular tubercle raised up; 3 pairs of lateral eyes; telotarsus with lateral margins ending in rounded lobes; *Est* located in distal of hand (Scorpionidae, see Stockmann & Ythier, 2010, p. 201); pedipalp femur with three trichobothria; patella of pedipalp with 19 trichobothria, three on ventral and 13 on external surface; chela of pedipalp with 26 trichobothria; retrolateral pedal spurs absent; lateroapical margins of tarsi produced into rounded lobes; metasomal segments I to IV with paired ventral submedian carinae; stridulatory organ located on opposing surfaces of pedipalp coxa and first leg; total length 60 to 180 mm	*Heterometrus* Ehrenberg, 1828
7	Chela trichobothrium *dt* present	*Liocheles* Sundevall, 1833
–	Chela trichobothrium *dt* absent	*Tibetiomachus* Lourenço & Qi, 2006

### Key to species of Family Chaerilidae from Xizang (China)

**Table:** 

1	Movable finger of pedipalp with 7–8 rows of granules	2
–	Movable finger of pedipalp with 10–14 rows of granules	6
2	Chela length to width ratio in adults 1.6–1.8	*Chaerilus conchiformus* Zhu, Han & Lourenço, 2008
2	Chela length to width ratio in adults higher than 2.0	3
–	Ventral side of seventh mesosomal segment with 2 pair of granular carina, anterior margin straight with a median notch	4
–	Ventral side of seventh mesosomal segment with many granules but without carina, anterior margin straight without median notch	5
4	Pedipalp femur shorter than carapace; 8–9 minute teeth on inner ventral margins of movable and immovable fingers respectively	*Chaerilus dibangvalleycus* Bastawade, 2006
–	Pedipalp femur longer than carapace, 7–8 minute teeth on inner ventral margins of movable and immovable fingers respectively	*Chaerilus mainlingensis* Di & Zhu, 2009
5	Manus of pedipalp in male narrow and long. Chela length/width ratio in male higher than 3	*Chaerilus tryznai* Kovařík, 2000
–	Manus of pedipalp in male robust. Chela length/width ratio in adults lower than 2.6	*Chaerilus wrzecionkoi* Kovařík 2012
6	Movable finger of pedipalp with 13–14 rows of granules; telson of male rather long and about 4.7 times longer than wide, with a obvious sexual dimorphism	*Chaerilus pictus* (Pocock, 1890)
–	Movable finger of pedipalp with 11–12 rows of granules, telson of male and female without sexual dimorphism, manus lacks 1 dorsal carina	7
7	Carapace, tergites nearly smooth in adults, chelicerae dorsal aspect without granules (Zhu, Han & Lourenço, 2008)	*Chaerilus tessellatus* Qi, Zhu & Lourenço, 2005
–	Carapace, tergites with many big granules in adults, chelicerae dorsal aspect with granules	*Chaerilus tricostatus* Pocock, 1899

### Key to species of family Euscorpiidae from Xizang (China)

**Table:** 

1	Trichobothrium *Eb_3_* on external surface of chela is located between trichobothria *Dt* and *Est*. Telson vesicle/aculeus juncture with annular ring (*Euscorpiops*)	2
–	Trichobothrium *Eb_3_* on the external aspect of pedipalp chela located basally from trichobothrium *Dt*. Annular ring at vesicle/aculeus juncture absent (*Scorpiops*)	5
2	Number of trichobothria on external surface of pedipalp patella: 19 (5 *eb*, 2 *esb*, 2 *em*, 5 *est*, 5 *et*)	3
–	Number of trichobothria on external surface of pedipalp patella: 17–18 (5 *eb*, 1–2 *esb*, 2 *em*, 4 *est*, 5 *et*)	4
3	Number of trichobothria on ventral surface of patella: 7; number of pectinal teeth: 4–5; movable finger longer than carapace and as long as pedipalp femur	*Euscorpiops kamengensis* Bastawade, 2006
–	Number of trichobothria on ventral surface of patella: 9; pectinal teeth number 8; movable finger as long as carapace and shorter than pedipalp femur	*Euscorpiops novaki* Kovarík, 2005
4	Female pedipalp fingers nearly straight	*Euscorpiops asthenurus* (Pocock, 1900)
–	Female pedipalp fingers obviously scalloped	*Euscorpiops karschi* Qi, Zhu &Lourenço, 2005
5	Fingers of pedipalps are straight or only slightly flexed in both sexes	6
–	Fingers of pedipalps are flexed (curved) in both sexes	7
6	Ventral trichobothria on patella number 6 (7 rarely), total length 30–42.1mm, pectinal teeth number 4–5, chela length to width ratio about 2.2	*Scorpiops jendeki* Kovařík, 2000 (Yunnan)
–	Ventral trichobothria on patella number 7, total length 40–58 mm, pectinal teeth number 7–9, chela length to width ratio about 3.3–3.5	*Scorpiops leptochirus* Pocock, 1893
7	Male chela length to width ratio about 1.8–2.2; the manus with same or very similar length and width, fingers of pedipalps are very strongly flexed in the male; ventral trichobothria on patella number 6–8	*Scorpiops hardwickii* (Gervais, 1843) “complex”
–	Male chela length to width ratio above 2.2; or the manus with length longer than width, or ventral trichobothria on patella number more than 8	8
8	Total length more than 65 mm	9
–	Total length less than 65 mm	10
9	Mostly yellowish to yellow in adults, ventral patella of pedipalps with 9 trichobothria	*Scorpiops luridus* Qi, Zhu & Lourenço, 2005
–	Mostly red brown in adults, ventral patella of pedipalps with 7 (rarely 6 or 8) trichobothria	*Scorpiops petersii* Pocock, 1893
10	Dorsally flat manus of pedipalps and chela of both sexes with length/width ratio: 2.1–2.2 (mean about 2.1 in males and 2.2 in females), total length 40.0–50.0 mm in adults	*Scorpiops margerisonae* Kovařík, 2000
–	Dorsally round manus of pedipalps or at least the chela of one sex with length to width ratio higher than 2.2 or total length higher than 50 mm	11
11	Total length more than 50 mm, chela strong, with length/width ratio: 2.0 in male and 2.5 in female	*Scorpiops tibetanus* Hirst, 1911
–	Total length less than 40 mm	12
12	Chela of pedipalp length to width ratio about 2.6–3.0, dorsal surface of chela of pedipalp coarse	*Scorpiops lhasa* Di & Zhu, 2009
–	Chela of pedipalp length to width ratio lower than 2.5, dorsal surface of chela of pedipalp smooth with luster	*Scorpiops atomatus* Qi, Zhu & Lourenço, 2005

#### Scorpiops jendeki

Kovařík, 2000

http://species-id.net/wiki/Scorpiops_jendeki

Figures 119 –135 , Table 3

Scorpiops jendeki Kovařík, 2000: 180, 182, figs 59–60, tabs 1–3.
Scorpiops hardwickii jendeki : Kovařík, 1994: 62, figs 7–13, tab. 1; Fet, 2000b: 492.
Scorpiops jendeki : Di et al., 2011b: 29–30, figs 118–122.

##### Type locality.

China, Yunnan, Gaoligongshan Nature Reserve 100 km west of Baoshan.

##### Type material.

Holotype, female, China, Yunnan, Gaoligongshan Nature Reserve 100 km west of Baoshan; 1 female paratype (NMPC), 4 females paratypes (FKCP),14–21/6/1993, E. Jendek and O. Sausa leg.

##### Material examined.

3 females and 1 immature male (MHBU, Ar.- MHBU- YNLL0801–4, 0804 is male), China, Yunnan Province, Baoshan City, Longling County, 7/2008, Ji-Shan Xu and Zhen-Hua Gao leg.

##### Diagnosis.

Total length is 30–42.1 mm. Patella with 17 external trichobothria (5 *eb*, 2 *esb*, 2 *em*, 4 *est*, 4 *et*) (Fig. 127) and 6–7 ventral trichobothria (6 specimens, Fig. 128). Pectinal teeth count 4–5. Both males and females have fingers of pedipalps straight, without any flexure. The carapace bears very sparse large granules.

*Scorpiops jendeki* appears to be closely related to *Scorpiops hardwickei* (Gervais, 1843), both species have the same number of external and ventral trichobothria on the patella, and a similar length/width ratio of chela; however, in the latter the fingers of pedipalps are strongly flexed.

##### Description.

(based on female specimen: Ar.- MHBU -YNMH0801).

*Coloration*: mainly yellow. Carapace red brown with yellow stripe. Median and lateral ocular tubercles black. Tergites mostly dark red brown to dark brown with yellow stripe. Metasoma segments dark red brown to dark brown. Vesicle red yellow brown with brown stripe and a red brown aculeus. Chelicerae yellow brown with fingers dark red brown gradually lighter toward the tip. Pedipalp femur and patella dark red brown, chela manus and fingers red brown. Legs red brown with yellow stripe, tarsi yellow brown. Tarsal ungues yellowish brown. Sternum, genital operculum and sternites pale brown. Pectines yellowish.

*Morphology*. *Prosoma*: Carapace with sparse, big granules (Fig. 120); anterior edge with big granules, lateral and posterior edges smooth; lateral furrow broad, anterior median furrow broad and moderately deep, posterior median furrow deep; margin behind lateral eyes with granules, other margins smooth. Median eyes situated anteriorly compared to center of carapace; three pairs of lateral ocelli, posterior smallest (Fig. 123). Median ocular tubercle smooth with a pair of median eyes which are much larger than lateral eyes, and a median furrow. Lateral ocular tubercle with some granules around eyes.

*Mesosoma*: Tergites sparsely covered with coarse granules, posterior part of tergites with bigger granules; tergites III‒VI with a median swell and two pairs of lateral carinae (shaped by bigger granules). Pectinal teeth count 4/4, fulcra absent (Fig. 124). Genital operculum subtriangular. Sternites smooth and shiny; segment VII with 4 smooth ventral carinae.

*Metasoma*: Tegument coarse. Segments II to V longer than wide; segments I to V with respectively 10-8-8-8-7 carinae; ventromedian, ventrolateral carinae stronger distally, dorsal carinae with small granules, lateral carinae weaker distally; segment V carinae with smaller granules dorsally and larger serration ventrally (Fig. 129). Vesicle with few setae and granules. Aculeus short and slightly curved (Fig. 130). The boundary between vesicle and aculeus not sharp.

*Pedipalps*: Tegument coarse. Femur with external, dorsointernal, dorsoexternal, ventrointernal, ventroexternal and internal carinae with round granules; tegument with few small granules dorsally (Fig. 125) and smooth ventrally. Patella (Figs 126–128) with dorsointernal, dorsoexternal, ventrointernal, ventroexternal and external carinae with round granules; two large spinoid granules present on the internal aspect; tegument with few granules dorsally and ventrally nearly smooth. Trichobothrial pattern C, neobothriotaxic (Vachon 1974); patella with 17 external trichobothria (5 *eb*, 2 *esb*, 2 *em*, 4 *est*, 4 *et*), 6 ventral trichobothria. Chela with length/width ratio: 2.2 in adult males and 2.2–2.4 in adult females (2.2 on female holotype and a male specimen in Kovařík 2000b: 160, tab. 1) (Figs 131–135). Chela with dorsal marginal, external secondary, and ventrointernal carinae granulated. For position and distribution of trichobothria on the tibia of pedipalp see (Figs 132–135).

*Chelicerae*: Tegument smooth. Movable finger with 4 teeth on dorsal edge, 4 teeth on ventral edge. Fixed finger with 3 teeth on dorsal edge (Figs 121, 122).

*Legs*: Tegument coarsely granular dorsally, except basitarsi and telotarsi, smooth ventrally. Femur dorsal surface with few small granules, external surface with a granular carina, internal surface with two granular carinae. Patella internally with a dentate carina. Tibia with few setae and small granules, without spurs. Basitarsi with some spinules, few setae and 2 lateral pedal spurs. Tarsi ventrally with one row of short spinules and few setae. Tarsal ungues curved and hook-like.

**Figure 119.**
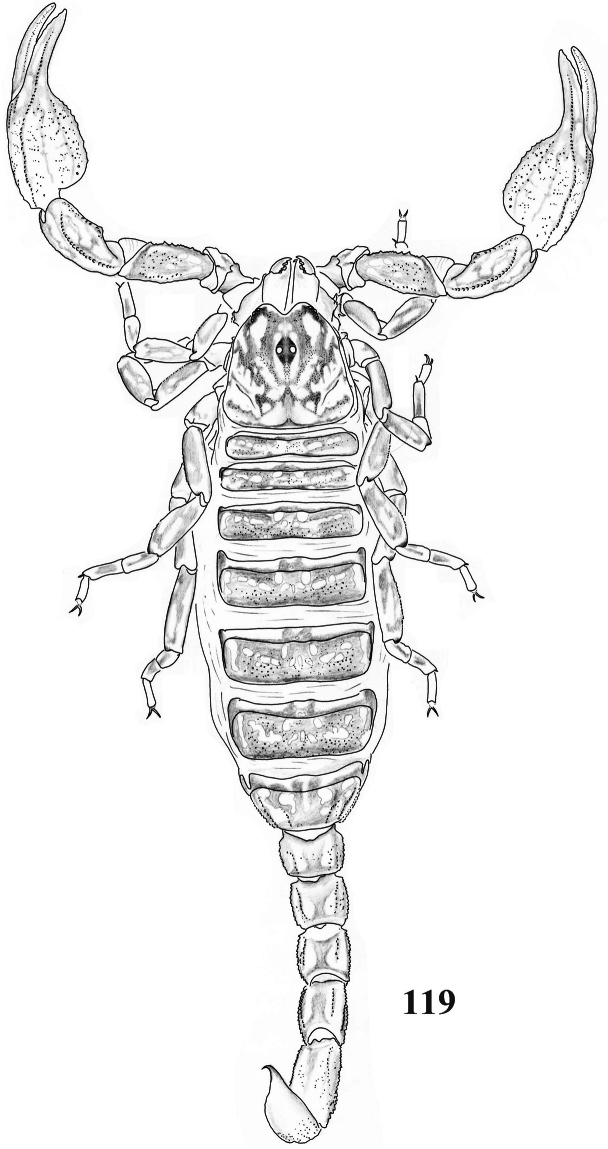
Habitus of *Scorpiops jendeki* from Longling County, female, dorsal view.

**Figures 120–131.**
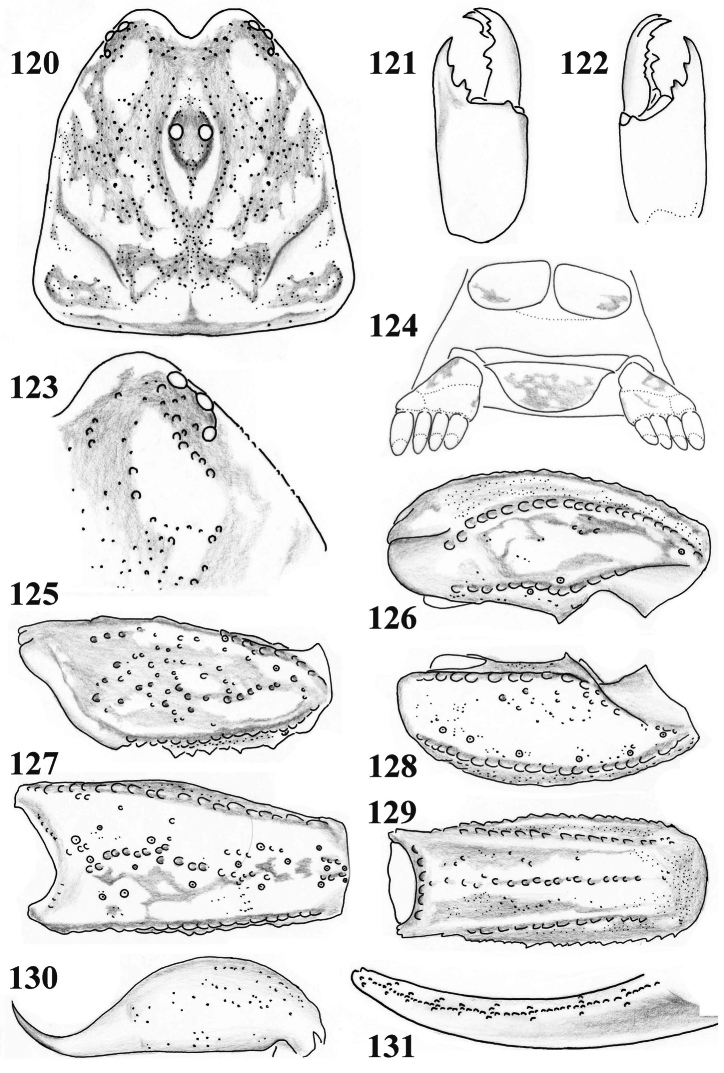
*Scorpiops jendeki* from Longling County, female. **120** Carapace **121–122** Chelicera, dorsal and ventral aspects **123** Lateral eyes **124** Genital operculum and pectines **125** Femur dorsal aspect **126–128** Patella dorsal, external and ventral aspects **129** Metasomal segment V, ventral aspect **130** Telson, lateral aspect **131** Dentate margin of movable finger, showing rows of granules.

**Figures 132–135.**
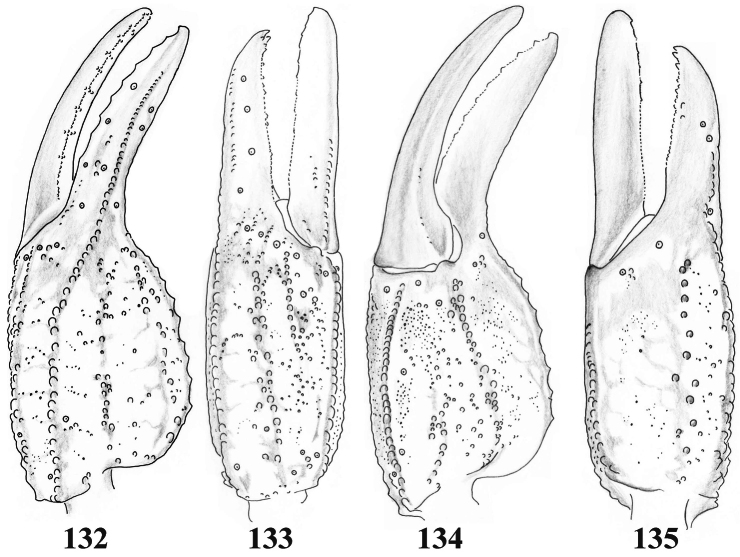
*Scorpiops jendeki* from Longling County, female. Chela dorsal, external, ventral and internal aspects.

##### Variation.

Female and male materials: coloration and morphology are very similar to holotype. Sexual dimorphism is not distinct. Total length is 30–42.1mm. 6–7 ventral trichobothria on the patella of pedipalps. Pectinal teeth count 4–5. Measurements in Table 3.

##### Ecology.

This species is uncommon, collected from moist mixed forest and in the bark or leavers and moss.

##### Distribution.

Yunnan (China).

## Discussion

**Figure 136–139.**
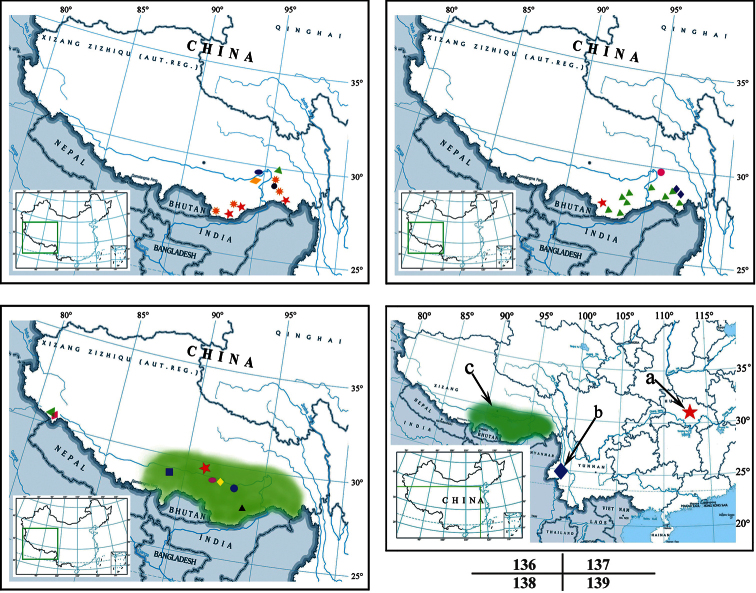
**136** Map of Xizang (China), showing the localities of the *Chaerilus* species. Map abbreviations: **a** (ellipse and rhombus) *Chaerilus conchiformus* **b** (round) *Chaerilus dibangvalleycus* **c** (rhombus) *Chaerilus mainlingensis* **d** (triangle and round) *Chaerilus tryznai* (macula) **e** (star) *Chaerilus tricostatus* **f** (pentagram) *Chaerilus pictus* **g** (triangle) *Chaerilus wrzecionkoi* **h** (ellipse, triangle and macula) *Chaerilus tessellatus*. The red line showing the scorpions appears to be restricted to latitude north of 31°N, bordered by Burang - Lhasa- Maizhokunggar - Gongbo’gvamda – Bomê line **137** Map of Xizang (China), showing the localities of Euscorpiops species. Map abbreviations: **a** (triangle) *Euscorpiops asthenurus* **b** (pentagram) *Euscorpiops kamengensis* **c** (rhombus) *Euscorpiops karschi* **d** (round) *Euscorpiops novaki* **138** Map of Xizang (China), showing the localities of Scorpiops species, Heterometrus tibetanus and Hottentotta songi. Map abbreviations: **a** (round) *Scorpiops atomatus*, *Scorpiops langxian* and *Scorpiops luroris* **b** (black triangle) *Scorpiops leptochirus* **c** (pentagram) *Scorpiops lhasa* **d** (ellipse) *Scorpiops margerisonae* **e** (square) *Scorpiops tibetanus* **f** (yellow rhombus) *Scorpiops pococki* **g** (purple rhombus) *Heterometrus tibetanus* **h** (green triangle), *Hottentotta songi* **139** Map of China, showing the localities of Scorpios species. Map abbreviations: **a** (pentagon), *Scorpiops* sp. from Hubei (Huzhaoshan Mountains) **b** (rhombus), *Scorpiops jendeki* from Yunnan (Gaoligongshan Mountains) **c** (green part), the area rich in *Scorpiops* (Xizang).

Twenty-six scorpion species of 7 genera and 5 families (Buthidae: *Hottentotta* (1 species), *Isometrus* (1); Chaerilidae: *Chaerilus* (8); Euscorpiidae: *Euscorpiops* (4), *Scorpiops* (10); Hemiscorpiidae: *Tibetiomachus* (1); Scorpionidae: *Heterometrus* (1)) were recorded in Xizang, all of them distribute in south and the north shores of Yarlung Zangbo Jiang: south of 31°N, bound on the north by the Burang - Lhasa- Maizhokunggar - Gongbo’gvamda - Bomê line (Figs 136–139). In them, 20 of 26 recorded species are endemic (76.9%).

In China, the closest area of scorpion fauna with Xizang is Yunan. Except one *Scorpiops* sp. was found in Hubei, all of euscorpiids were found in Xizang and Yunnan. Species of the genera *Scorpiops* and *Euscorpiops* are dominant, with confined distribution and not overlapped in Xizang and Yunnan. All of the species of family Chaerilidae found in China are living in Xizang. Qinghai, Sichuan and Xinjiang, are also with border on of Xizang. In Qinghai, just *Mesobuthus martensii martensii* (Karsch, 1879) reported in its northeast (Zhu et al., 2004; Zhang & Zhu, 2009). There is no scorpion species reported in Sichuan (Zhu et al., 2004). In Xinjiang, species genera of the family Buthidae recorded (*Mesobuthus* (7 species and subspecies), *Razianus* (1)) (Zhu et al., 2004; Lourenço et al., 2010; Sun and Sun, 2011). *Mesobuthus martensii martensii* (Karsch, 1879) and *Mesobuthus eupeus mongolicus* (Birula 1911) found in South of Gansu which also belong to Qinghai-Tibetan Plateau (Sun and Sun, 2011). We conjecture the vast area of gap of scorpion distribution in the north of Xizang and the south of Qinghai is caused by the cold and clammy climate. So the scorpion fauna of Xizang isn’t related to Qinghai and Xinjiang.

In the world, the 7 genera found in Xizang were recorded distributing to the south of Xizang. Modern species of genera *Chaerilus*, *Euscorpiops* and *Scorpiops* are limited to tropical areas of South Asia and Southeast Asia, although they reached considerable altitudes in Kashmir, Nepal, and Tibet (Kovařík, 2000a, 2000b). The distribution of the species of genera *Hottentotta*, *Isometrus*, *Heterometrus* and the close related genera of *Tibetiomachus* also suggest the scorpion fauna of Xizang is close to South Asia and Southeast Asia.

## Supplementary Material

XML Treatment for Hottentotta songi

XML Treatment for Isometrus (Reddyanus) tibetanus

XML Treatment for Chaerilus conchiformus

XML Treatment for Chaerilus dibangvalleycus

XML Treatment for Chaerilus mainlingensis

XML Treatment for Chaerilus pictus

XML Treatment for Chaerilus tessellatus

XML Treatment for Chaerilus tricostatus

XML Treatment for Chaerilus tryznai

XML Treatment for Chaerilus wrzecionkoi

XML Treatment for Euscorpiops asthenurus

XML Treatment for Euscorpiops kamengensis

XML Treatment for Euscorpiops karschi

XML Treatment for Euscorpiops novaki

XML Treatment for Scorpiops atomatus

XML Treatment for Scorpiops hardwickii

XML Treatment for Scorpiops langxian

XML Treatment for Scorpiops leptochirus

XML Treatment for Scorpiops lhasa

XML Treatment for Scorpiops luridus

XML Treatment for Scorpiops margerisonae

XML Treatment for Scorpiops petersii

XML Treatment for Scorpiops pococki

XML Treatment for Scorpiops tibetanus

XML Treatment for Tibetiomachus himalayensis

XML Treatment for Heterometrus tibetanus

XML Treatment for Scorpiops jendeki

## References

[B1] Bai M (2004) Chapter 1: Natural environment and resources assessment. In: Bai M (Eds). Xizang Geography. The Peoples Press of Xizang, Lhasa, China: 6-92.

[B2] Bastawade DB (2006) Arachnida: Scorpionida, Uropygi, Schizomida and Oncopodid Opiliones (Chelicerata). Zool. Surv, India. Fauna of Arunachal Pradesh, State Fauna Series 13 (2): 449-465.

[B3] Di ZY He YW Cao ZJ Wu YL Li WX (2011a) The first record of the family Euscorpiidae (Arachnida: Scorpiones) from Central China, with a key of Chinese species of the genus *Scorpiops*. Euscorpius 116: 1-11.

[B4] Di ZY He YW Wu YL Cao ZJ Liu H Jiang DH Li WX (2011b) The scorpions of Yunnan (China): updated identification key, new record and redescriptions of *Euscorpiops kubani* and *E. shidian* (Arachnida, Scorpiones). ZooKeys 82: 1-33. doi: 10.3897/zookeys.82.715 PMC3088492 21594054

[B5] Di ZY Cao ZJ Wu YL Zhu L Liu L Li WX (2013) The scorpions of Hainan Island, China (Arachnida: Scorpiones) Euscorpius (In press).

[B6] Di ZY Wu YL Cao ZJ Fan LQ Li WX (2009) The genus *Chaerilus* Simon, 1877 (Scorpiones: Chaerilidae) in China, with a description of the female *C. tricostatus* Pocock, 1899. Arthropoda Selecta 18 (3/4): 131−138.

[B7] Di ZY Wu YL Cao ZJ Xiao H Li WX (2010) A catalogue of the genus *Euscorpiops* Vachon, 1980 (Scorpiones: Euscorpiidae, Scorpiopinae) from China, with description of a new species. Zootaxa 2477: 49−61.

[B8] Di ZY Zhu MS (2009a) One new species of the Genus *Scorpiops* Peters, 1861 (Scorpiones: Euscorpiidae, Scorpiopinae) from Xizang, China. Zootaxa 2030: 39−48.

[B9] Di ZY Zhu MS (2009b) A new species of *Chaerilus* Simon, 1877 (Scorpiones, Chaerilidae) from China. Acta Arachnologica 58 (2): 97−102. doi: 10.2476/asjaa.58.97

[B10] Di ZY Zhu MS (2009c) The male of *Euscorpiops karschi* (Scorpiones: Euscorpiidae, Scorpiopinae) from China (Xizang). Arthropoda Selecta 18 (1/2): 9−16.

[B11] Di ZY Zhu MS (2010) Redescription of *Scorpiops margerisonae* Kovařík, 2000, with the first record of its female, from China (Scorpiones: Euscorpiidae: Scorpiopinae). Euscorpius 104: 1−9.

[B12] Fet V (2000a) Family Chaerilidae Pocock, 1893. In: Fet, V., Sissom, W. D., Lowe, G. & Braunwalder, M. E., Catalog of the Scorpions of the World (1758–1998). The New York Entomological Society, New York, 323–328.

[B13] Fet V (2000b) Family Ischnuridae Simon, 1879. In: Fet V Sissom WD Lowe G Braunwalder ME (Eds). Catalog of the Scorpions of the world (1758–1998). The New York Entomological Society, New York: 383-408.

[B14] Fet V (2000c) Family Scorpionidae Latreille, 1802. In: Fet V Sissom WD Lowe G Braunwalder ME (Eds). Catalog of the Scorpions of the world (1758–1998). The New York Entomological Society, New York: 427-486.

[B15] Fet V (2000d) Family Scorpiopidae Kraepelin, 1905. In: Fet V Sissom WD Lowe G Braunwalder ME (Eds). Catalog of the Scorpions of the World (1758–1998). The New York Entomological Society, New York: 487-495.

[B16] Fet V Lowe G (2000) Family Buthidae C. L. Koch, 1837. In: Fet V Sissom WD Lowe G Braunwalder ME (Eds). Catalog of the Scorpions of the world (1758–1998). The New York Entomological Society, New York: 54-286.

[B17] Fet V Bechly G (2001) Case 3120a. Liochelidae, fam. nov. (Scorpiones): proposed introduction as a sustitute name for Ischnuridae Simon, 1879, as an alternative to the suggested emendment of Ischnurinae Fraser, 1957 (Insecta, Odonata) to Ischnurainae in order to remove homonymy. Bull. Zool. Nomen. 58 (4): 280-281.

[B18] Fet V Sissom WD (2000) Family Euscorpiidae Laurie, 1896. In: Fet V Sissom WD Lowe G Braunwalder ME (Eds). Catalog of the Scorpions of the world (1758–1998). The New York Entomological Society, New York: 355-380.

[B19] Hirst S (1911) Descriptions of new scorpions. Annals and Magazine of Natural History, 8 (8): 462–473. doi: 10.1080/00222931108693056

[B20] Hjelle JT (1990) Anatomy and morphology, in: G.A. Polis (Ed.), The Biology of Scorpions. Stanford Univ. Press, pp. 9–63.

[B21] Kishida K (1939) “Arachnida of Jehol. Scorpiones”. Report of the first scientific expedition to Mandchoukuo under the leadership of Shigeyasu Tokunage. June–October 1933, 5, 1, 4 (10): 1–66.

[B22] Kishida K (1939) Arachnida of Jehol. Order Scorpiones. Report of the First Scientific Expedition to Manchoukuo Under the Leadership of Shigeyasu Tokunaga, June–October 1933, 5, 1 (4), 10: 49–67.

[B23] Kovařík F (2000a) Revision of family Chaerilidae (Scorpiones),with descriptions of three new species. Serket 7 (2): 38-77.

[B24] Kovařík F (2000b) Revision of family Scorpiopidae (Scorpiones),with descriptions of six new species.Acta Soc. Zool. Bohem 64: 153-201.

[B25] Kovařík F (2003) A review of the genus *Isometrus* Ehrenberg, 1828 (Scorpiones: Buthidae) with descriptions of four new species from Asia and Australia. Euscorpius 10: 1-19.

[B26] Kovařík F (2004) A review of the genus *Heterometrus* Ehrenberg, 1828, with descriptions of seven new species (Scorpiones, Scorpionidae). Euscorpius 15: 1-60.

[B27] Kovařík F (2005) Three new species of the genera *Euscorpiops* Vachon, 1980 and *Scorpiops* Peters, 1861 from Asia (Scorpiones: Euscorpiidae, Scorpiopinae). Euscorpius 27: 1-10.

[B28] Kovařík F (2007) A revision of the genus *Hottentotta* Birula, 1908, with descriptions of four new species (Scorpiones: Buthidae). Euscorpius 58: 1-107.

[B29] Kovařík F Ahmed Z (2009) Three new species of *Scorpiops* Peters, 1861 (Scorpiones: Euscorpiidae: Scorpiopinae) from Pakistan. Euscorpius 88: 1-11.

[B30] Kovařík F (2012a) *Euscorpiops thaomischi* sp. n. from Vietnam and a key to species of the genus (Scorpiones: Euscorpiidae: Scorpiopinae). Euscorpius 142: 1−8.

[B31] Kovařík F (2012b) Five new species of *Chaerilus* Simon, 1877 from China, Indonesia, Malaysia, Philippines, Thailand, and Vietnam (Scorpiones: Chaerilidae). Euscorpius 149: 1−14.

[B32] Lamoral BH (1980) A reappraisal of supragenic classification of recent scorpions and their zoogeography. Proc. 8th Int. Congr. Arachnol. Vienna 1980: 439−444.

[B33] Lourenço WR Duhem B (2010) The genus *Chaerilus* Simon, 1877 (Scorpiones, Chaerilidae) in the Himalayas and description of a new species. Zookeys 37: 13-25. doi: 10.3897/zookeys.37.369

[B34] Lourenço WR Duhem B Leguin EA (2011) The genus *Chaerilus* Simon, 1877 (Scorpiones, Chaerilidae) in the Indian Ocean Islands and description of a new species. Euscorpius 110: 1−8.

[B35] Lourenço WR Qi JX (2006) Mountain scorpions: a new genus and species from Tibet (China). C R Biol Apr; 329 (4): 289-295. 10.1016/j.crvi.2006.02.005 16644501

[B36] Lourenço WR Qi JX Zhu MS (2005) Description of two new species of scorpions from China (Tibet) belonging to the genera *Mesobuthus* Vachon (Buthidae) and *Heterometrus* Ehrenberg (Scorpionidae). Zootaxa 985: 1-16.

[B37] Lourenço WR Sun D Zhu MS (2010) *Razianus xinjianganus* sp. nov.: A new record Genus and new species of (Scorpiones, Buthidae) from China. Journal of Hebei University (Natural Science Edition) 30 (3): 307-312.

[B38] Lourenço WR Zhu MS (2008) A new species of the genus *Isometrus* Ehrenberg, 1828 (Scorpiones, Buthidae) from China. Acta Zootaxonomica Sinica, 33 (2): 264–271.

[B39] Monod L Volschenk ES (2004) *Liocheles litodactylus* (Scorpiones: Liochelidae): An unusual new *Licheles* species from the Australian wet tropics (Queensland). Memoirs of the Queensland Museum, 49 (2): 675-687.

[B40] Prendini L (2000) Phylogeny and classification of the superfamily Scorpionoidea Latreille, 1802 (Chelicerata, Scorpiones): An exemplar approach. Cladistics 16: 1-78. doi: 10.1111/j.1096-0031.2000.tb00348.x 34902920

[B41] Qi J X Zhu MS Lourenço WR (2005) Eight new species of the genera *Scorpiops* Peters, *Euscorpiops* Vachon, and *Chaerilus* Simon (Scorpiones: Euscorpiidae, Chaerilidae) from Tibet and Yunnan, China. Euscorpius 32: 1-40.

[B42] Shi CM Huang ZS Wang L He LJ Hua YP Leng L Zhang DX (2007) Geographical distribution of two species of *Mesobuthus* (Scorpiones, Buthidae) in China: insights from systematic field surveys and predictive models. The Journal of Arachnology 35: 215-226. doi: 10.1636/T06-20.1

[B43] Sissom WD (1990) Systematics, biogeography and paleontology. In: Polis, G.A. (ed.). The Biology of Scorpions. Stanford University Press, Stanford, California. pp.64–160.

[B44] Soleglad ME Fet V (2003) High–level systematics and phylogeny of the extant scorpions (Scorpiones: Orthosterni). Euscorpius 11: 1-175.

[B45] Soleglad ME Fet V Kovařík F (2005) The systematic position of the scorpion genera *Heteroscorpion* Birula, 1903 and *Urodacus* Peters, 1861 (Scorpiones: Scorpionoidea). Euscorpius 20: 1-38.

[B46] Soleglad ME Sissom WD (2001) Phylogeny of the family Euscorpiidae Laurie, 1896: a major revision. In: Fet V, Selden PA (Eds) Scorpions 2001. In Memorium Gary A. Polis. Brit. Arachnol. Soc. pp 25–111.

[B47] Sun D Sun, ZN (2011) Notes on the genus *Mesobuthus* (Scorpiones: Buthidae) in China, with description of a new species. The Journal of Arachnology 39: 59-75. doi: 10.1636/Ha10-36.1

[B48] Sun D Zhu MS Lourenço WR (2010) A new species of *Mesobuthus* (Scorpiones: Buthidae) from Xinjiang, China, with notes on *Mesobuthus songi*. The Journal of Arachnology 38: 35-43. doi: 10.1636/Ha09-39.1

[B49] Teruel R Rein JO (2010) A new species of *Hottentotta* Birula, 1908 from Afghanistan, with a note on the generic position of *Mesobuthus songi* Lourenço, Qi et Zhu, 2005 (Scorpiones: Buthidae). Euscorpius 94: 1−8.

[B50] Vachon M (1952) *Etudes sur les scorpions*. Publications de l’Institut Pasteur d’Algérie, Alger, 482 pp. 18864077

[B51] Vachon M (1973) [1974] Etude des caractères utilisés pour classer les familles et les genres de Scorpions (Arachnides). 1. La trichobothriotaxie en arachnologie. Sigles trichobothriaux et types de trichobothriotaxie chez les Scorpions. Bulletin du Muséum national d’Histoire naturelle, Paris, 3e sér. , 140: 857-958.

[B52] Zhang L Zhu MS (2009) Morphological variation of *Mesobuthus martensii* (Karsch, 1879) (Scorpiones: Buthidae) in Northern China. Euscorpius 81: 1-17.

[B53] Zhu MS Han GX Lourenço WR (2008) The chaerilid scorpions of China (Scorpiones: Chaerilidae). Zootaxa 1943: 37-52.

[B54] Zhu MS Qi JX Song DX (2004) A Checklist of Scorpions from China (Arachnida: Scorpiones). Acta Arachnologica Sinica 13 (2): 111-118.

